# Safety and Efficacy of Adoptive Transfer of Stem Cell Memory Enriched Virus Specific T Cells against CMV and EBV

**DOI:** 10.1002/advs.202510288

**Published:** 2025-12-08

**Authors:** Xun‐Hong Cao, Xu‐Ying Pei, Yuan‐Yuan Zhang, Juan Xie, Jing‐Wei Tu, Zhuo‐Jun Liu, Yi‐Yang Ding, Chen‐Hua Yan, Yu‐Hong Chen, Yu Wang, Lan‐Ping Xu, Xiao‐Hui Zhang, Xiao‐Jun Huang, Xiang‐Yu Zhao

**Affiliations:** ^1^ Peking University People's Hospital, Peking University Institute of Hematology National Clinical Research Center for Hematologic Disease Beijing Key Laboratory of Cell and Gene Therapy for Hematologic Malignancies Peking University Beijing 100044 China; ^2^ Shanghai iCELL Biotechnology Co., Ltd Shanghai Engineering Research Center of Stem Cells Translational Medicine Shanghai 200335 China; ^3^ Institute for Regenerative Medicine State Key Laboratory of Cardiology and Medical Innovation Center Shanghai East Hospital School of Life Sciences and Technology Tongji University Shanghai 200123 China; ^4^ Peking‐Tsinghua Center for Life Sciences Academy for Advanced Interdisciplinary Studies Peking University Beijing China

**Keywords:** CMV, EBV, hemopoietic stem cell transplantation, stem cell‐like memory, virus‐specific T cells

## Abstract

Adoptive immunotherapy with third‐party virus‐specific T lymphocytes (VSTs) is effective against refractory viral infections. However, its long‐term efficacy and persistence must be enhanced. T memory stem cells (TSCMs) with superior self‐renewal and multilineage differentiation potential may enhance VSTs durability, although their antiviral capacity is underexplored. Cytomegalovirus (CMV)‐and Epstein–Barr virus (EBV)‐specific T cells are enriched with CD8⁺ TSCM through cytokine and peptide stimulation. Comprehensive preclinical evaluations show that purified TSCM‐VSTs exhibit reduced exhaustion, enhanced expansion, and stronger antiviral activity than central or effector memory VSTs (TCM or TEM). Transcriptomic and epigenetic analyses show significant enrichment of the MAPK and Wnt signaling pathways, consistent with stem‐like characteristics. In a murine model, CD8⁺ TSCM VSTs provide more effective protection against Raji‐pp65 tumors than TCM or TEM VSTs. In a phase I clinical trial, 10 patients with refractory CMV or EBV infections post‐transplant who received third‐party, off‐the‐shelf TSCM‐enriched VSTs show a 100% overall response rate and 70% complete response, with persistence up to 12 weeks and no severe adverse events. These findings support TSCM‐enriched VSTs as a potent, scalable antiviral immunotherapy and highlight TSCM proportion as a critical determinant of VSTs efficacy.

## Graphical abstract

This study finds that CD8⁺ TSCM cells exhibit superior self‐renewal, differentiation, and antiviral activity. Transcriptome and epigenome analyses highlight MAPK cascade regulation in TSCM cells. In vivo, virus‐specific TSCM cells show enhanced persistence and tumor protection. Clinically, phase I trial data indicate a 100% overall response rate, supporting the therapeutic potential of third‐party, allogeneic CMV‐ and EBV‐specific TSCMs.

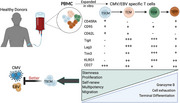

## 1 Introduction

Individuals with immunodeficiency and recipients of hematopoietic stem cell transplantation (HSCT) often exhibit compromised immune function and a paucity of virus‐specific immune cells.^[^ ^1^, ^2^, ^3^ ^]^ This immunodeficiency predisposes them to the reactivation of latent viruses, such as cytomegalovirus (CMV) and Epstein‐Barr virus (EBV), leading to concurrent viral infections and subsequent organ damage. Notably, CMV and EBV co‐infections, whether simultaneous or sequential, are frequently observed in these patient populations and are associated with adverse clinical outcomes.^[^ ^4^, ^5^ ^]^ Although prophylactic and preemptive antiviral therapies have reduced the incidence of these infections, their efficacy remains suboptimal due to their limited effectiveness and associated toxicities.^[^ ^6^ ^]^

Adoptive transfer of virus‐specific T lymphocytes, derived either from stem cell donors or third‐party sources, has been increasingly used to prevent and treat viral infections after HSCT. Early studies have demonstrated that donor‐derived virus‐specific T lymphocytes (VSTs) could effectively control CMV, EBV, and adenovirus infections, with response rates exceeding 70% in some cohorts.^[^ ^7^, ^8^, ^9^ ^]^ Subsequent trials using banked third‐party VSTs have expanded access to patients lacking suitable donors, showing rapid antiviral effects and low incidence of graft‐versus‐host disease (GVHD).^[^ ^10^, ^11^ ^]^ Despite these successes, challenges remain, including the limited persistence of the transferred cells, mostly composed of TEM subsets, and the delayed onset of efficacy in some patients.

Memory T cells, particularly T memory stem cells (TSCMs), exhibit self‐renewal and multilineage differentiation potential, making them promising candidates for conferring durable immunity against viral infections.^[^ ^12^, ^13^ ^]^ TSCMs are characterized by elevated expression of transcription factors, such as TCF1, LEF1, and Eomes, and have been shown to persist for decades in humans, as observed in recipients of the yellow fever vaccine.^[^ ^14^ ^]^ Recent studies have highlighted the potential role of TSCM and TCM subsets in enhancing VST persistence and function.^[^ ^15^, ^16^, ^17^ ^]^ Palianina et al. reported that in vitro‐expanded EBV‐specific TSCMs provided long‐term control of EBV‐driven tumors in preclinical mouse models, demonstrating the potential of TSCM to enhance the efficacy of adoptive T‐cell therapies.^[^ ^15^ ^]^ However, the contribution of different virus‐specific T cell memory subsets (TSCM, TCM, and TEM) to their antiviral efficacy has not been systematically compared, which is a key focus of the current investigation. Moreover, the safety and efficacy of TSCM‐enriched VST have not been explored in clinical settings.

In the present study, we successfully expanded clinical‐grade virus‐specific CD8⁺ TSCM‐enriched VSTs in vitro using cytokines and viral peptides from CMV (pp65 and IE1) and EBV (LMP2a and EBNA1). We performed a preclinical study to characterize the features and antiviral efficacy of TSCM VSTs compared to those of TCM and TEM VSTs, both in vitro and in mice. We also conducted a phase I clinical trial involving 10 patients to assess the therapeutic potential of third‐party, off‐the‐shelf TSCM‐enriched VSTs.

## 2 Results

### 2.1 TSCMs Expanded from PBMCs Using IL2, IL4, IL7, IL12, IL15 Cytokines and CMV and EBV Peptides Displayed Virus‐Specific Responses

**Figure 1:**
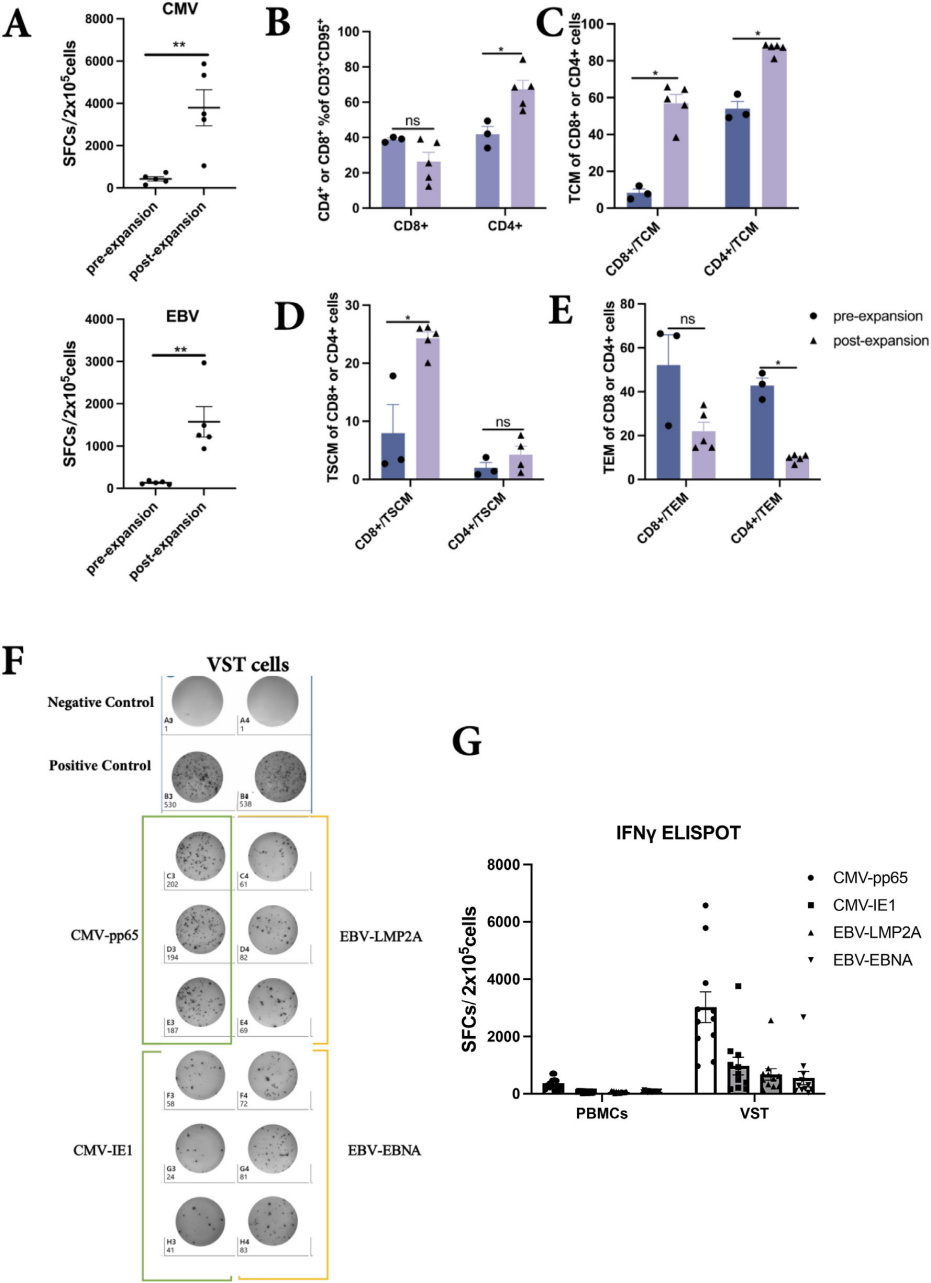
Virus‐specific T cells enriched with high percentages of TSCM and TCM expanded successfully in vitro. A) Detection of CMV and EBV‐specific T cells in pre‐expanded PBMCs and post‐expanded VST products by IFN‐γ ELISPOT assay. B–E) Proportion of T cell subsets pre (n = 3) and after expansion (n = 5) (Mann‐Whitney test); F) IFN‐γ ELISpot assay for monitoring anti‐CMV and EBV performance in expanded VSTs cells, unstimulated VSTs were used as a negative control. G) SFC counts in IFN‐γ ELISpot following stimulation of unexpanded PBMCs (plated at 2 × 10E5 cells per well) and VSTs cells (plated at 5 × 10E4 cells per well) with CMV and EBV peptides (n = 10).

Following a 2‐week expansion of PBMCs using IL2, IL4, IL7, IL12, IL15 cytokines and CMV (pp65 and IE1) and EBV (LMP2a and EBNA1) peptides, the final product achieved an average 13‐fold (11.12–15.82) expansion (Figure S1B, Supporting Information), resulting in ≥ 99% CD3^+^CD95^+^ virus‐specific T cells (data not shown). Moreover, IFN‐γ ELISPOT assays demonstrated that CMV‐specific T cells expanded ≈132.5‐fold (52.9–203.2) and EBV‐specific T cells expanded 143.1‐fold (71.3–249.7) (**Figure** **1**A).

Notably, there was a significant increase in the proportion of CD4⁺ T cells (% of CD3⁺CD95⁺), while no statistically significant change was observed in CD8⁺ T cells (Figure 1B, Figure S1C, Supporting Information) among CD3+ T cells. Further analysis of CD8⁺ and CD4⁺ T cell subsets based on CD45RA and CD62L expression revealed that the expanded CD8⁺ T cell population was primarily composed of TSCM (CD45RA+CD62L+) and TCM (CD45RA−CD62L+) subsets, whereas the expanded CD4⁺ T cell population was predominantly composed of TCM subsets (Figure 1C,D). Remarkably, the absolute number of CD8⁺ TSCMs increased ≈1000‐fold (480–1600) during expansion, demonstrating the robust selective enrichment of this critical memory subset (Figure S1D, Supporting Information). Correspondingly, the percentages of TEM cells in both CD4⁺ and CD8⁺ T cell populations significantly decreased post‐expansion (Figure 1E, Figure S1E, Supporting Information), indicating the successful amplification of TSCMs in the VST products.

The specificity of the expanded VST products was assessed using an IFN‐γ enzyme‐linked immunospot (ELISpot) assay targeting EBV and CMV antigens. The results demonstrated that VST products effectively recognized both CMV and EBV antigens compared with unexpanded PBMCs, with spot‐forming cells (SFCs) exceeding 300 per 2 × 10⁵ cells compared to the negative control. Additionally, the frequency of T cells responsive to CMV (IE1 and pp65) was higher than that of T cells responsive to EBV (EBNA1 and LMP2A) (Figure 1F,G).

### 2.2 Virus‐Specific CD8+TSCMs displayed lower exhaustion and Multifaceted Antiviral Capacity Signature than CD8+TCM Cells In Vitro

**Figure 2:**
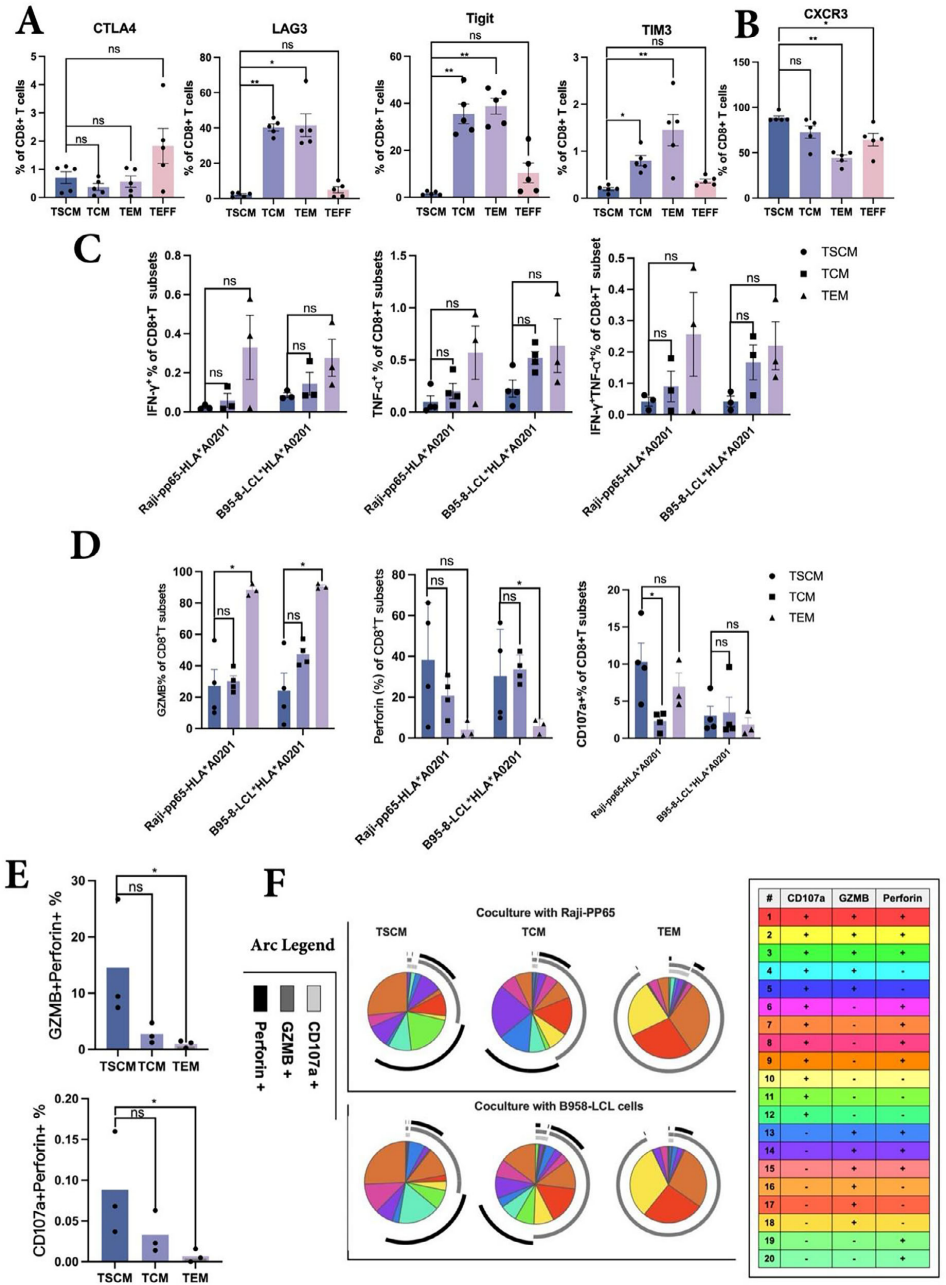
Virus‐specific CD8+TSCM display a less pronounced effector signature compared with CD8+TCM cells in vitro. A, B) Exhaustion molecules and CXCR3 expression of different virus‐specific memory T cells (n = 5) (Kruskal‐Wallis test). C) Assessment of IFN‐γ, TNF‐α as well as IFN‐γ+ TNF‐α+ secretion in purified CD8+T cell subsets after co‐cultured with different cell lines (n = 3–4 per group, Kruskal‐Wallis test). D) Assessment of GZMB, Perforin, CD107a in purified CD8+T cell subsets after co‐cultured with different cell lines (n = 3–4 per group, Kruskal‐Wallis test). E) Expression of GZMB+Perforin+(%of CD8+T subsets) when cocultured with Raji‐pp65‐HLA*A0201 cells(up) and expression of CD107a+Perforin+(% of CD8+T subsets) when cocultured with EBV‐LCLs (Bottom). F) Display of polyfunctional cytotoxic molecules (CD107a, GZMB and Perforin) among CD8+T subsets (%) when cocultured with Raji‐pp65‐HLA*A0201(Upper) and B958‐LCL cells (Lower) (n = 3–4 per group).

The effector functions of CD8⁺ T cells are influenced by various factors, including surface activation, inhibitory receptor expression, and maturation status. Our findings indicated that CD8⁺ TSCMs within the VST products exhibited significantly lower expression of LAG‐3, TIGIT, and TIM‐3 than TCM and TEM, although differences relative to TEFF cells were not statistically significant (**Figures** **2**A, S2A, Supporting Information). Although CTLA‐4 protein levels did not show significant differences at the protein level, transcriptomic profiling revealed reduced CTLA‐4 expression in CD8⁺ TSCMs relative to other subsets (Figure S2B, Supporting Information). These results suggest that CD8⁺ TSCMs constitute a population with reduced exhaustion at both the phenotypic and transcriptomic levels.

Furthermore, TSCMs displayed significantly lower levels of the senescence‐associated marker, KLRG1, although CD57 expression remained comparable across subsets. In contrast, CD27, a co‐stimulatory molecule associated with early memory and proliferative potential, was significantly upregulated in TSCMs relative to TCM and TEM cells, reinforcing their stem‐like, less differentiated status (Figure S2C, Supporting Information). Additionally, the antigen‐specific activation marker CD137 was significantly less prevalent in CD8⁺ TSCMs than in other subsets, whereas CD69 expression remained unchanged (Figure S2D, Supporting Information).

We sorted different virus‐specific CD8⁺ T cell subsets and co‐incubated them with Raji‐pp65‐HLA*A0201 and EBV‐LCLs to assess their cytotoxicity and cytokine production against CMV and EBV. The results demonstrated that CD8⁺ TSCMs exhibited IFN‐γ, TNF‐α, and IFN‐γ⁺TNF‐α⁺ secretion levels comparable to those of CD8⁺ TCM and CD8⁺ TEM cells, with no significant differences observed among the subsets (Figure 2C). These findings suggest that CD8+TSCMs retain their effector functions upon antigen stimulation, although their advantages may not be prominent in this short‐term co‐culture assay.

Importantly, cytotoxic granule components, such as granzyme B(GZMB) and perforin, are key effector molecules that mediate the T cell–driven clearance of virus‐infected cells. TEM cells exhibited the highest GZMB expression under both CMV and EBV antigen stimulation. Although TSCMs showed relatively higher perforin levels than TEM cells under EBV antigen stimulation, there were no significant differences in CD107a or GZMB expression when compared to TEM cells (Figure 2D). Nevertheless, analysis of the combinatorial distribution across subsets revealed that TSCMs contributed a larger fraction to the GZMB⁺Perforin⁺ intersecting region of the arc‐shaped pie chart than TEM cells when co‐cultured with Raji‐pp65‐HLA*A0201 and exhibited a higher proportion of CD107a⁺Perforin⁺ cells when co‐cultured with EBV‐LCLs (Figure 2E,F), indicating their multifaceted antiviral capacity. This granule‐based effector function complements previously observed cytokine‐mediated responses, reflecting the diverse antiviral strategies employed by different CD8⁺ T cell subsets.

### 2.3 Virus‐specific CD8+ TSCM Displayed Stem Cell‐Like Molecular Characteristics

**Figure 3:**
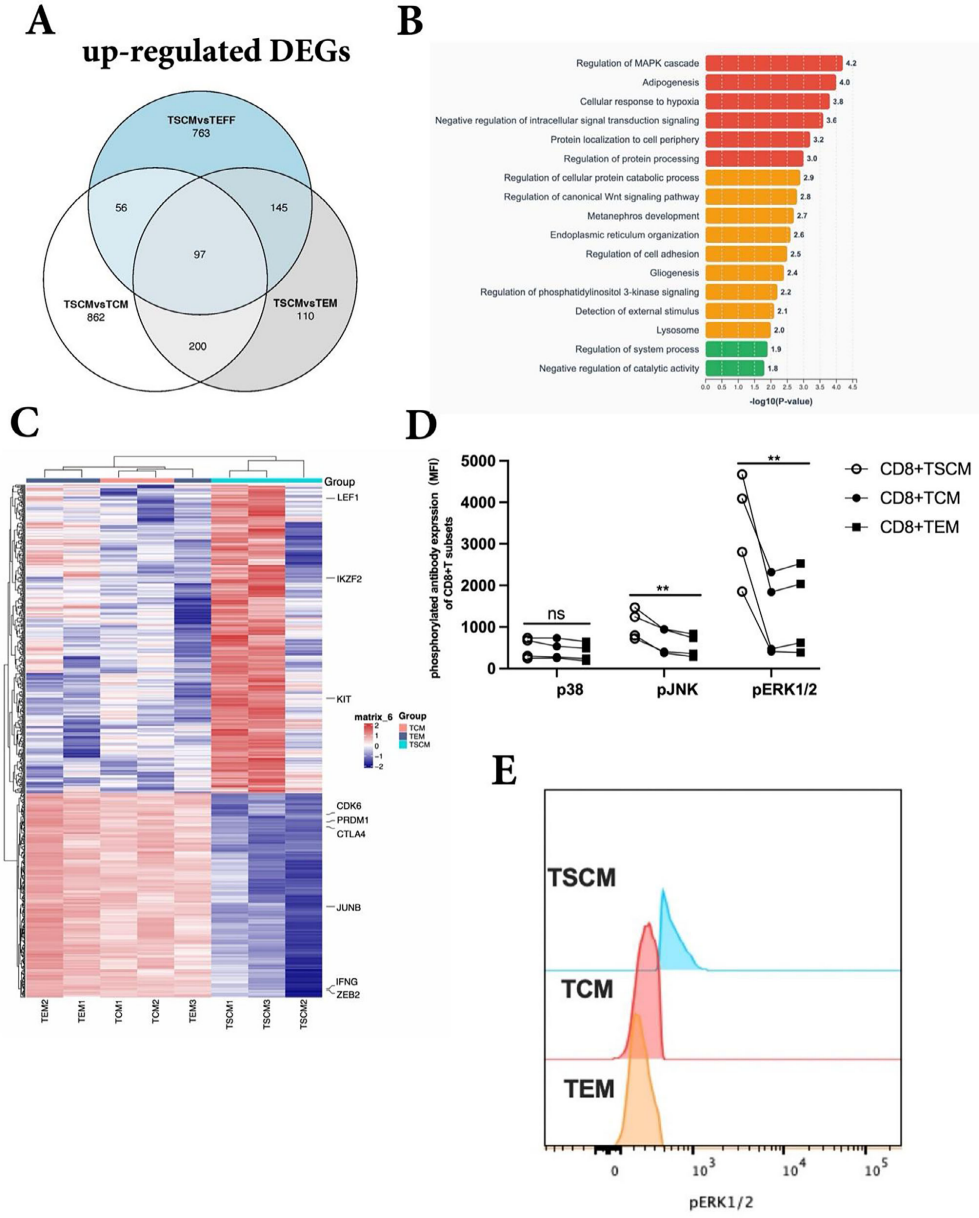
Virus‐specific CD8+TSCM displayed stem cell like molecular characteristics. A) After comparing the two groups of TEM, TCM and TEFF with CD8+TSCMs, the up‐regulated differential genes were intersected via Venn. B) Go enrichment analysis of upregulated intersection genes via Metascape website. C) Comparison of chromatin opening differences among different CD8+T cell subsets; D) Differences in the expression of p38, pJNK and pERK1/2 of different CD8+T cell subsets (n = 4.group, Wilcoxon paired t‐test); E) A typical flow cytometry diagram of pERK1/2 in CD8+T subsets.

To explore the molecular signatures of virus‐specific CD8+TSCMs compared with those of TCM and TEM cells, we used bulk RNA‐seq to evaluate gene and pathway differences. Principal component (PC) analysis demonstrated that the CD8+TSCM group had similar characteristics to the TCM group in PC1, but separately from TEM cells (Figure S3A, Supporting Information). We then compared the differential genes in CD8+TCM/TEM/TEFF versus TSCMs pairwise and merged the upregulated and downregulated genes shared between each group using a Venn diagram. We identified 97 differentially expressed genes (DEGs) and 106 downregulated DEGs among the groups. Gene ontology (GO) enrichment pathway analysis found that some upregulated genes were enriched in the GO term “regulation of canonical Wnt signaling pathway” (**Figure** **3**A,B, Figure S3B, Supporting Information), consistent with a previous study, which proved that Wnt signaling is important for TSCM self‐renewal and differentiation ability.^[^ ^18^ ^]^

Although RNA‐seq captures steady‐state gene expression levels among virus‐specific CD8+TSCMs versus TCM and TEM cells, it does not directly reflect the underlying regulatory potential of chromatin. To address this, we performed ATAC‐seq, which revealed key differences in chromatin accessibility among the T subsets. Notably, loci associated with stemness‐related transcription factors, such as LEF1, IKZF2, and KIT, showed increased openness in CD8⁺ TSCMs, whereas effector‐related factors, including JUNB, ZEB2, and PRDM1, were more accessible in CD8⁺ TEM and TCM cells (Figure 3C). These epigenetic differences, which were not fully captured by transcriptome profiling alone when we integrated RNA‐seq‐derived differentially expressed genes (DEGs) and ATAC‐seq–identified differentially accessible regions (DARs) between TSCM and TCM cells, showed consistent upregulation of stemness‐associated genes, such as LEF1 and FOXP1, in both datasets. However, some transcription factors with known roles in stemness (e.g., KIT and IKZF2) or effector differentiation (e.g., JUNB and PRDM1) did not show consistent patterns across the RNA‐seq and ATAC‐seq datasets (Figure S3C, Supporting Information). These results provide a critical context for the observed transcriptional heterogeneity and support the notion that cell‐intrinsic chromatin states underlie the functional divergence of T cell subsets.

Consistent with this, transcriptome analysis further demonstrated that virus‐specific CD8⁺ TSCMs highly expressed stemness‐ and memory‐associated transcription factors (TCF7, FOXO1, ID3, and BCL6), as well as homing and adhesion molecules (CCR7 and SELL) (Figure S3D,E, Supporting Information). In addition, pathway analysis revealed that “regulation of MAPK cascade” was enriched most in the upregulated genes, indicating an important role for MAPK signaling in regulating VST‐CD8+TSCMs (Figure 3A,B). Flow cytometry further confirmed the functional relevance, showing significantly higher expression of pERK1/2 in CD8+TSCMs than in TCM and TEM cells, whereas phosphorylated p38 and pJNK showed no significant differences (Figure 3D,E). Taken together, these findings indicate that distinct chromatin architectures, in concert with transcriptional programs, govern the lineage identity and antiviral potential of virus‐specific memory CD8⁺ T cell subsets.

### 2.4 Virus‐specific CD8+ TSCM Displayed Enhanced Self‐Renewal and Multipotency In Vitro and In Vivo

**Figure 4:**
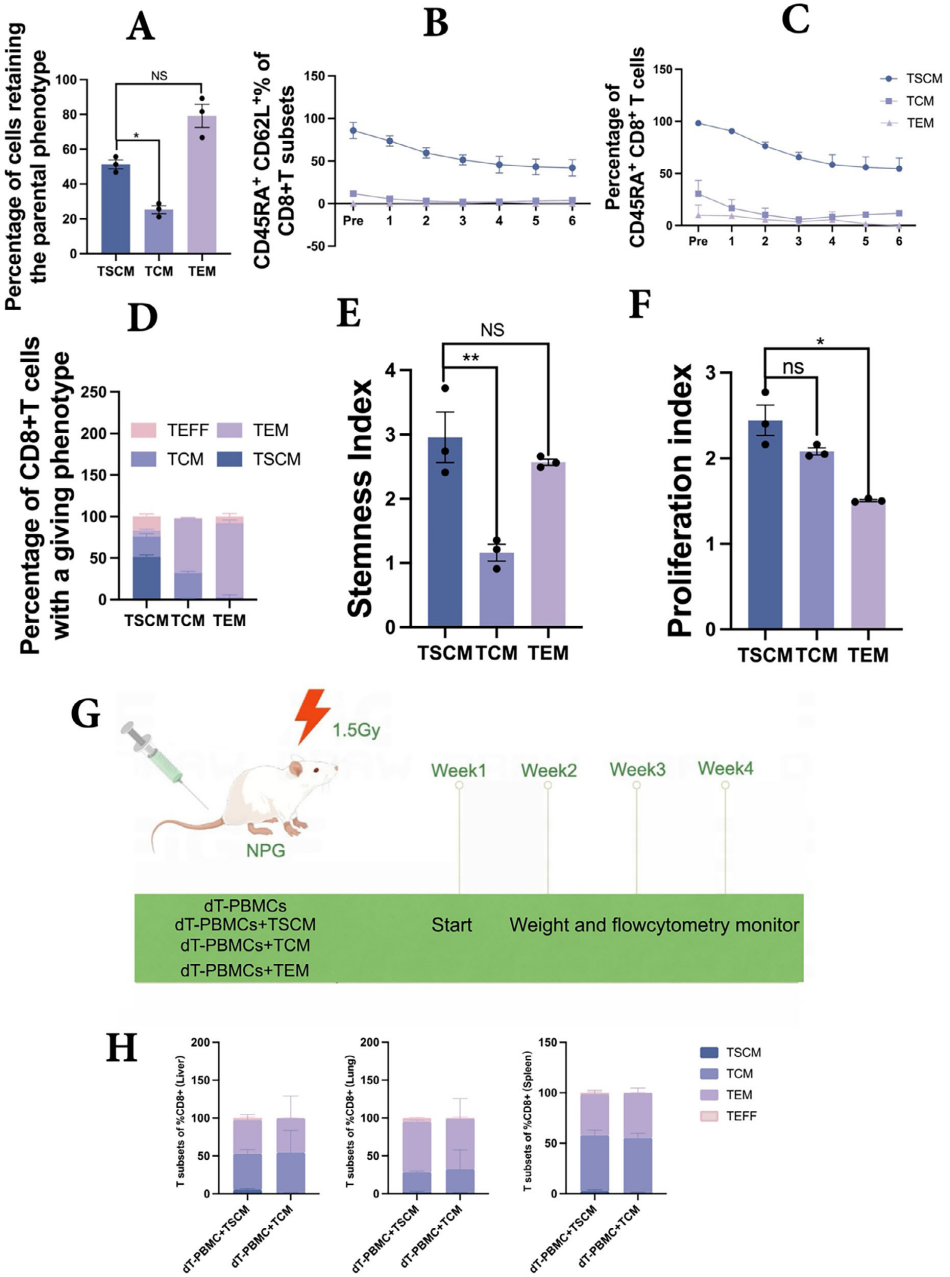
Enhanced self‐renewal and multipotency of Virus‐specific CD8+TSCMs. A) The proportion of CD8+T cell subsets that maintain the parental phenotype (n = 3, Kruskal‐Wallits test). B,C) Differences in CD45RA+CD62L+ and CD45RA+ expression among diverse CD8+T cell subsets at different proliferation passages(n = 3). D) Differentiation level of CD8+T cell subsets after 5‐days of CD3/CD2/CD28 stimulation in vitro(n = 4). E) Assessment of stemness index of CD8+T subsets (n = 3, Kruskal‐Wallits test. F) Assessment of proliferation index of CD8+T subsets after 5‐days of CD3/CD2/CD28 stimulation in vitro (n = 3, Kruskal‐Wallis test). G) Diagram of the colonization detection model of CD8+T cells infused into NPG mice, mice were injected with 1 × 10⁶ cells per T cell subset, with a 1:1 ratio of dT‐PBMCs to each T cell subset; H) The proportion of T cell differentiation subsets in mouse liver, lung and spleen after infusion of different T cell subpopulations for 4 weeks (n = 6 per group, independent two experiments).

We further evaluated the self‐renewal and differentiation capacities of virus‐specific CD8⁺ TSCMs in comparison with those of TCM and TEM cells. After stimulation with anti‐CD3/CD2/CD28 for 5 days, ≈50% of CD8⁺ TSCMs retained the CD45RA (CD62L) phenotype as well as CD45RA^+^ expression levels, demonstrating their ability to maintain the original phenotype, a hallmark of self‐renewal. As TSCM proliferation progressed, these dynamic phenotypic changes resulted in diverse progeny, with ≈20% differentiation into TCM cells, 5% into TEM cells, and 10% into TEFF cells, suggesting that TSCMs can reconstitute the entire T cell compartment. In contrast, as TCM proliferation progressed, TCM cells primarily retained the TCM phenotype or differentiated into TEM cells, whereas TEM cells largely maintained their original phenotype (**Figure** **4**A–D).

We further assessed the stemness of different subsets using the stem cell index,^[^ ^12^ ^]^ which confirmed that TSCMs exhibited significantly higher stemness scores than the TCM (Figure 4E). Despite their superior stemness, the proliferative capacity of TSCMs was comparable to that of TCM (Figure 4F, Figure S4A,B, Supporting Information).

Following the infusion of different virus‐specific T cell subsets into irradiated NPG mice, a higher proportion of human lymphocytes (hCD45%) was observed in mice receiving TSCM infusion than in those receiving TCM cells at 4 weeks post‐infusion. This trend was consistent across peripheral blood (PB) and tissues, including the spleen, liver, and lungs (Figure S4C,D, Supporting Information). CD8⁺ TSCMs differentiated into TCM, TEM, and TEFF subsets, whereas CD8⁺ TCM cells primarily gave rise to TEM and TCM subsets at 4 weeks post‐infusion in the liver, lungs, and spleen of the recipient mice (Figure 4G,H).

Taken together, these findings suggest that virus‐specific CD8⁺ TSCMs exhibit superior self‐renewal and multi‐lineage differentiation capacity compared to CD8⁺ TCM and CD8⁺ TEM cells.

### 2.5 Virus‐Specific CD8+ TSCM Displayed Decreased Apoptosis Induced by Virus Damage and Sustainable Antiviral Ability In Vitro

**Figure 5:**
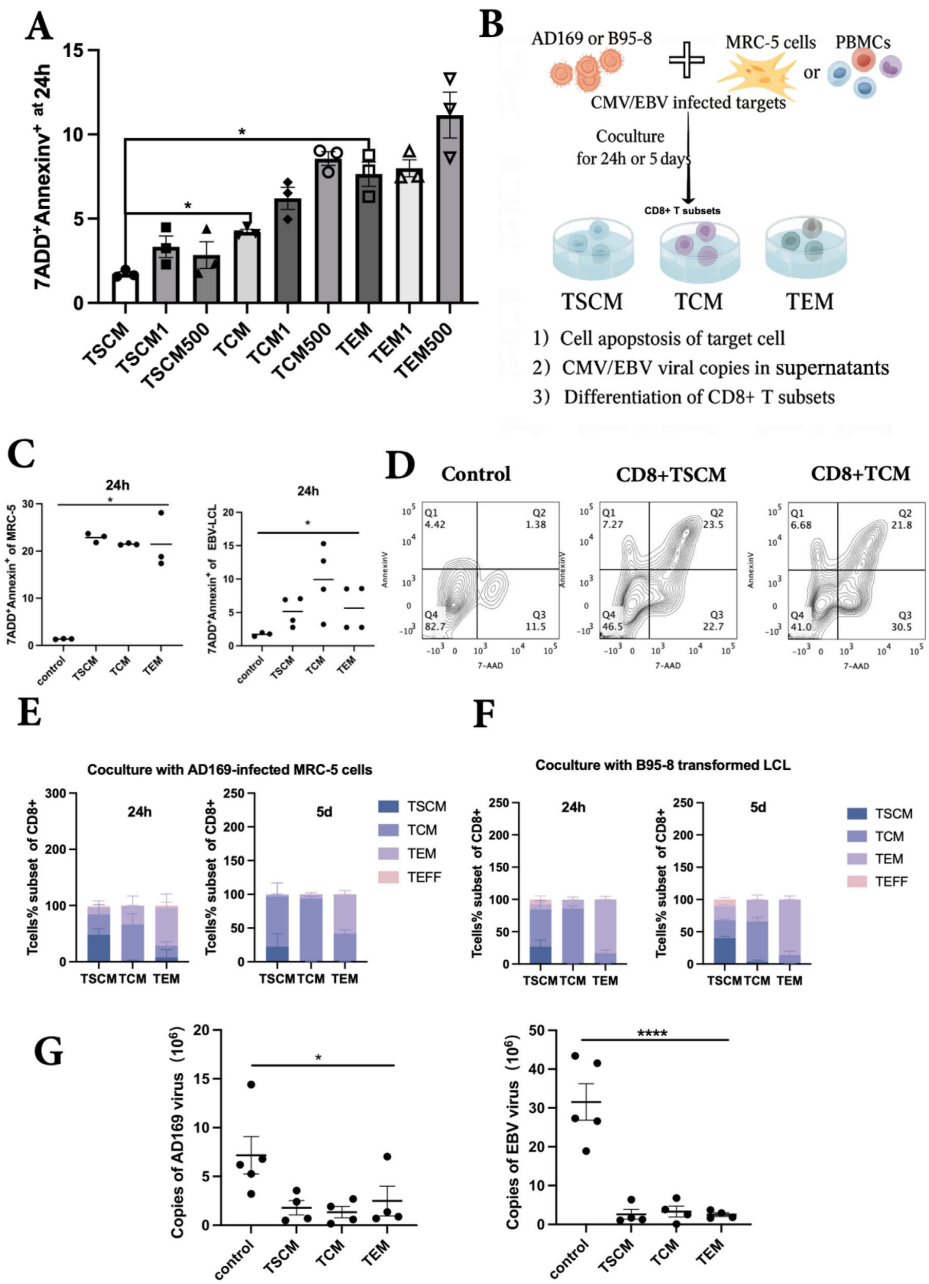
Decreased apoptosis and sustainable anti‐CMV and EBV effect in vitro. A) Differences in T cell apoptosis when incubated with AD169 virus strains of different MOI titers for 24 h (n = 3, Mann‐Whitney test). B) Model diagram for evaluating the antiviral functions of different CD8+T subsets in vitro. C) AD169‐MRC‐5 cells(left) or EBV‐LCLs(right) were cocultured with or without purified CD8+T cell subsets for 24 h and then the target cell apoptosis was assessed (n = 3 per group, Kruskal‐Wallis test), the control group is the target cells without cocultured CD8+T subsets. D) Diagram of typical flow cytometry for assessment of target cell apoptosis levels after co‐incubation of each CD8+T cell subset with AD169 infected MRC‐5 cells. E,F) Purified CD8+T cell subset was incubated with virus (CMV or EBV)‐infected target cells for 24h or 5‐days and then the cell differentiation state was evaluated (n = 4 per group). G) Different CD8+T cell subsets were incubated with CMV‐ and EBV‐transformed target cells for 5 days, and then the viral copies in the culture supernatant was evaluated (n = 4–5 per group, Kruskai‐Wallis test).

Multiple studies have shown that co‐culture with either virus‐infected or virally modified target cells, or in some cases, direct exposure to virus particles, can trigger apoptosis in CD4⁺ and CD8⁺ T cells through activation‐induced or virus‐mediated mechanisms.^[^ ^19^ ^]^ To further study whether the antiviral ability of TSCMs persisted longer than that of TCM and TEM cells, we first evaluated the apoptosis of different T cell subsets stimulated by AD169 virus at different MOI and found that the apoptosis rate of CD8+TSCM components was lowest with no more than 5% even in high concentration titers compared to TCM or TEM components (**Figure** **5**A) after 24 h of AD169 virus stimulation, although there is no significant difference after 48 h stimulation (Figure S5A), suggesting that TSCMs have a strong ability to resist virus damage, which may potentially lead to stronger sustained antiviral effects.

To address the potential concern of alloreactivity, we conducted a 4‐h PI‐staining cytotoxicity assay by co‐culturing VSTs with autologous or allogeneic PBMCs across a range of effector‐to‐target (E:T) ratios (1:1 to 10:1) to assess potential cross‐reactivity. The results clearly demonstrated that the VSTs did not exhibit significant cytotoxicity against allogeneic targets (Figure S5D, Supporting Information). Based on this, we evaluated the apoptosis level of AD169‐MRC‐5 cells or EBV‐LCLs with the HLA‐A*0201 locus to determine whether CD8+TSCMs exert a stronger specific antiviral effect (Figure 5B). The results showed that AD169‐MRC‐5 cells co‐incubated with CD8+TSCM had a tendency for higher apoptotic levels than those with CD8+TCM cells, although these differences did not reach statistical significance; comparable effects were observed among CD8+ memory T subgroups against EBV‐LCLs (Figure 5C,D). We next investigated whether this effect resulted from direct CD8+T cell activity or the influence of other effector T cells differentiating downward after encountering antigen stimulation from early differentiated memory CD8+T cells, thus indirectly exerting an antiviral effect. To prove this, we simultaneously analyzed the differences in differentiation among CD8+ T subsets after coculturing with AD169‐MRC‐5 or EBV‐LCL cells for 24 h. The results showed that the CD8+ TSCM group still maintained ≈48% and 38% of the CD45RA+CD62L+ phenotype, respectively, when co‐cultured with CMV‐ or EBV‐infected targets (Figure 5E,F).

We also used a 5‐day in vitro infection model to evaluate the effect of viral infection on T cell differentiation and the change in viral copy number after long‐term co‐culture of different cell subsets. We found that sorted CD8+TSCM significantly reduced CMV and EBV copy numbers compared to the control group, while there was no significant difference between TCM and TEM cells (Figure 5G). Further analysis of the differential ability of T cell subpopulations revealed that the CD8+TSCM group was dominated by TCM and TEM cells after 5‐day stimulation but still maintained about 22–34% of the TSCM phenotype when co‐cultured with AD169‐MRC‐5 cells and EBV‐LCLs separately (Figure 5E,F, Figure S5B, Supporting Information). Additionally, the proportion of alive CD3+ T cells was higher in the CD8+TSCM group than in the TEM groups after 5‐day co‐culture with AD169‐MRC‐5 cells (Figure S5C, Supporting Information), indicating the long‐lasting survival ability of CD8+TSCMs. In summary, CD8+ TSCMs exhibited sustained antiviral potential against CMV and EBV in vitro.

### 2.6 Virus‐Specific CD8+ TSCM Displayed Enhanced Anti‐CMV Ability In Vivo

**Figure 6:**
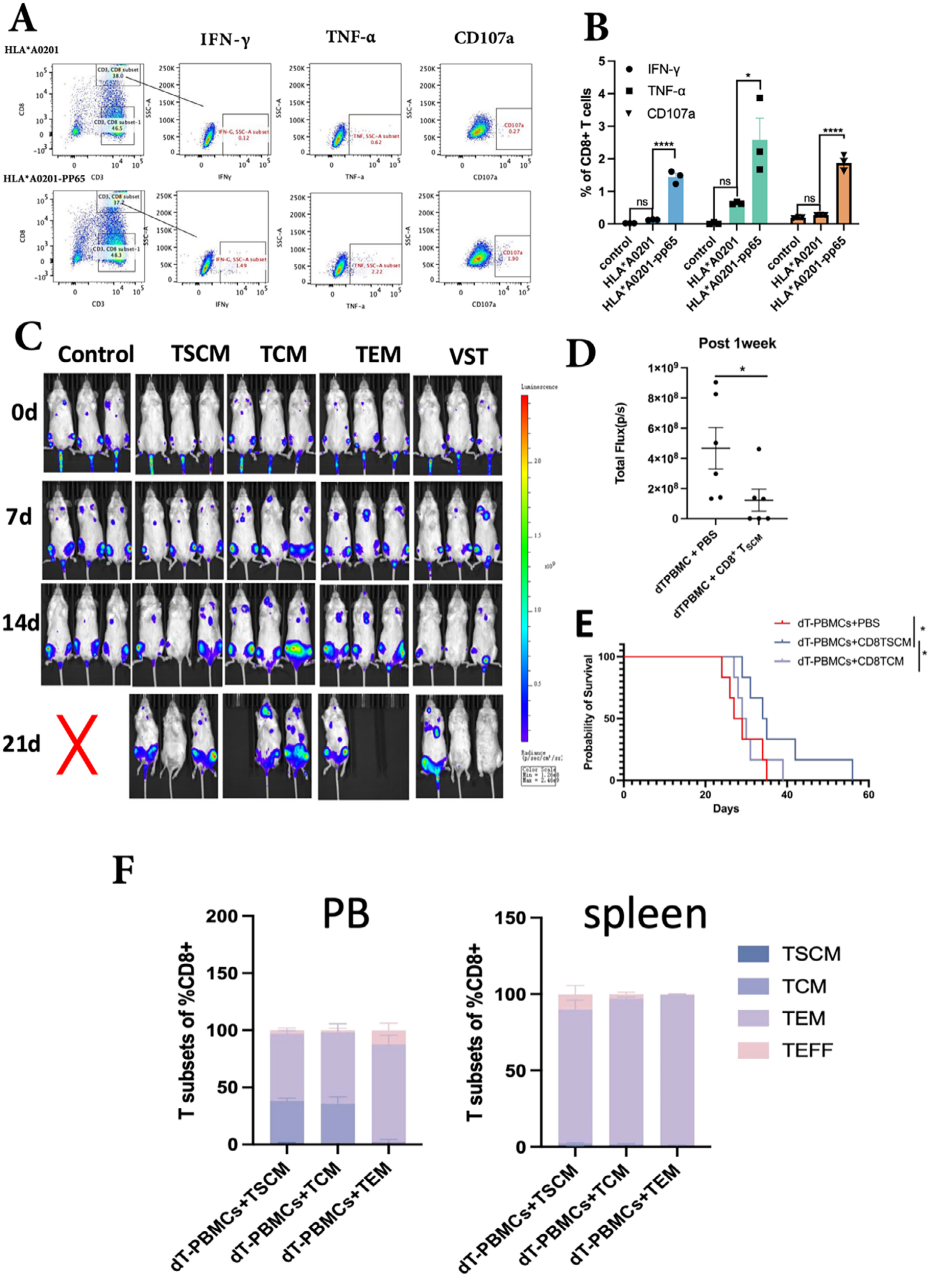
Virus‐specific CD8+TSCM displayed enhanced anti‐CMV ability in vivo. A) The typical flow cytometry distribution of IFN‐γ, TNF‐α and CD107a when VSTs cells were co‐incubated with Raji‐HLA*A‐0201 cell line with or without lentiviral transduced pp65 peptide. B) The expression of IFN‐γ, TNF‐α and CD107a in CD8+ T cells when co‐cultured with the Raji cells as the control group, Raji cells transduced with HLA*A0201 or Raji cells transduced with HLA*A0201 and pp65 (n = 3 per group, Wilcoxon paired **t**‐test). C, D) Assessment of tumor bioluminescence signal intensity in vivo before and after T infusion, the control group refers to mice that received only CD3‐depleted PBMCs (dT‐PBMCs) without any VSTs infusion; E) Survival evaluation after infusion of different CD8+T cell subsets (n = 6 per group, two independent experiments). F) Difference in the distribution of T cell subpopulations in peripheral blood (PB) and spleen 3‐weeks after infusion of different CD8+T cells in the subcutaneous tumor‐bearing model.

We constructed a tumor‐infiltrating mouse model by intravenously injecting Raji‐HLA*A0201‐pp65‐luciferase‐GFP cells to assess the in vivo activity of virus‐specific CD8+TSCMs (Figure S6A, Supporting Information). To confirm that the secretion of CD107a, IFNγ, and TNFα by VSTs was pp65 antigen specific rather than anti‐tumor effect, we co‐incubated VSTs with Raji‐HLA*A0201 cells, either with lentivirus‐transduced pp65 peptide or untransduced, for 5 h. Significantly higher levels of CD107a, IFN‐γ, and TNFα were observed only in the presence of pp65‐expressing Raji‐HLA*A0201 cells, indicating that the activation of VSTs was peptide‐specific (**Figure** **6**A,B). This indicated that the killing effect on Raji‐HLA*A0201‐pp65 cells was due to the activity of VSTs directly targeting the pp65 peptide.

In vivo tumor‐bearing experiments showed that in contrast to control mice without virus‐specific CD8+TSCM infusion, bioluminescence and tumor burden decreased over 1 week in mice treated with CD8+TSCMs at a dose of 1 × 10^6^ cells per mouse. The survival time of the CD8+TSCM infusion group was significantly longer than the PBS and the TCM infusion groups (Figure 6C–E).

Considering that different tumor seeding pathways may also affect adoptive cell efficacy, we constructed a subcutaneous Raji‐HLA*A0201‐PP65 model to evaluate the different efficacies of CD8+T cell subsets and whole VSTs. The results also showed that mice that received CD8+TEM cells exhibited higher tumor weights and volumes than the other groups after 3 weeks of infusion (Figure S6B–D, Supporting Information).

We also assessed the in vivo persistence of T cells to examine whether adoptively transferred CD8+TSCMs persisted better in vivo. Post 3‐week infusion, the adoptively transferred CD8+TSCM group showed better peripheral persistence with higher percentages of CD3‐CD8+T cells than the CD8+TCM and TEM groups (p = 0.0075, Figure S6E, Supporting Information) in the vein model. Similar trends were observed in the subcutaneous model, although the differences were not statistically significant (Figure S6F, Supporting Information). The differentiation ability of CD8+T cells in peripheral blood in the vein and spleen in the subcutaneous model showed that CD8+TSCM and CD8+TCM could reconstitute TEM and TEFF cells regardless of PB or spleen. There was no obvious difference between CD8+TSCM and CD8+TCM in the reconstructed TEM and TEFF subpopulations, indicating strong self‐renewal and differentiation abilities of early differentiated virus‐specific memory CD8+ T cells (Figure 6F, Figure S6G, Supporting Information).

### 2.7 Clinical Responses and Outcomes of Off‐The‐Shelf VSTs Enriched with TSCM Against CMV/EBV Infection

We presented clinical data from 10 patients in three dose groups (**Table** **1**), with four patients receiving the first dose of 1 × 10^6^ m^−2^, three patients receiving the second dose of 2 × 10^6^ m^−2^, and three patients receiving the third dose of 5 × 10^6^ m^−2^. Of these, four patients had refractory CMV infections or CMV disease, and six patients had refractory EBV PTLD. Off‐the‐shelf VSTs from three third‐party donors (Tables S1 and S2, Supporting Information) were administered between days 59 and 270, and two patients received T cell infusion after secondary HSCT.

**Table 1:** Clinical information of VSTs recipients.

Patient No.	Sex	Age, y	Diagnosis	Type of transplant	CMV IgG	EBV IgG	CMV/EBV Infection	Previous therapy	VSTs infusion day post HSCT	VSTs donor	VSTs dose cells/m^2^	Number of VSTs infusions	Class I HLA alleles match	CMV ELISPOT SFC/2 × 10^5^ cells	EBV ELISPOT SFC/2 × 10^5^ cells	Viral clearance(days)	Response at 28 day	6 months outcomes
1	Female	45	B‐ALL	Haplo	D+/R+	D+/R+	EBV PTLD	4 dose CD20	152	VST‐1	1 × 10E7	2	2	3923	698	7	PR	Alive
2	Male	24	AML	Haplo	D+/R+	D+/R+	Ref CMV	GCV for 3 weeks	239	VST‐1	1 × 10E7	2	2	3923	698	7	CR	Alive
3	Male	44	AML	Haplo (second)	D‐/R+	D+/R+	Ref CMV CMV Pneumonia	GCV for 2 months	270	VST‐3	1 × 10E7	2	2	2780	1055	7	CR	Alive
4	Male	54	T‐ALL/LBL	Haplo	D+/R+	D+/R+	EBV PTLD	4 dose CD20	116	VST‐1	1 × 10E7	2	2	3923	698	3	CR	Alive
5	Male	56	SAA	Haplo	D+/R+	D+/R+	EBV PTLD	2 dose CD20	60	VST‐2	2 × 10E7	2	1	1984	753	2	CR	Died
6	Female	38	AML	Haplo	D+/R+	D+/R+	EBV PTLD	2 dose CD20	59	VST‐2	2 × 10E7	2	1	1984	753	21	PR	Died
7	Male	30	AML	Haplo (second)	D+/R+	D+/R+	EBV PTLD	2 dose CD20	67	VST‐3	2 × 10E7	2	1	2780	1055	7	PR	Alive
8	Female	64	B‐ALL	Haplo	D‐/R+	D+/R+	Ref CMV	GCV for 2 weeks	168	VST‐1	5 × 10E7	2	1	3923	698	14	CR	Died
9	Female	30	HL	Haplo	D+/R+	D+/R+	EBV PTLD	2 dose CD20	62	VST‐3	5 × 10E7	2	1	2780	1055	3	CR	Alive
10	Male	51	BPDCN	Haplo	D‐/R+	D+/R+	Ref CMV	GCV for 2 weeks	151	VST‐2	5 × 10E7	2	1	1984	753	3	CR	Alive

**Abbreviations**: ALL, acute lymphoblastic leukemia; AML, acute myeloid leukemia; T‐ALL/LBL, T‐cell Acute Lymphoblastic Leukemia/Lymphoblastic Lymphoma; SAA, severe aplastic anemia; HL, Hodgkin Lymphoma; BPDCN, Blastic plasmacytoid dendritic cell neoplasm; Haplo, haploidentical stem cell transplantation; Ref, refractory; CMV, cytomegalovirus; EBV, Epstein‐Barr virus; D+/R+, donor serological positive/recipient serological positive; D‐/R+, donor serological negative/recipient serological positive; PR, partial remission; CR, complete remission.

**Note**: ELISPOT data represent the pre‐infusion quality control results for each batch of VST products; thus, patients receiving the same batch have identical values.

In general, adoptive therapy with VSTs was safe and well tolerated, with no immediate adverse reactions or cytokine release syndrome related to infusion within 42 days post infusion. No de novo aGVHD was observed, and two patients with prior grade 1–2 aGVHD were stable and did not require additional therapy after VSTs. No transfusion‐related hematologic toxicities (e.g., neutropenia, thrombocytopenia, or anemia) or new immunosuppressive therapy requirements were triggered post‐infusion (Table S3, Supporting Information).

**Figure 7:**
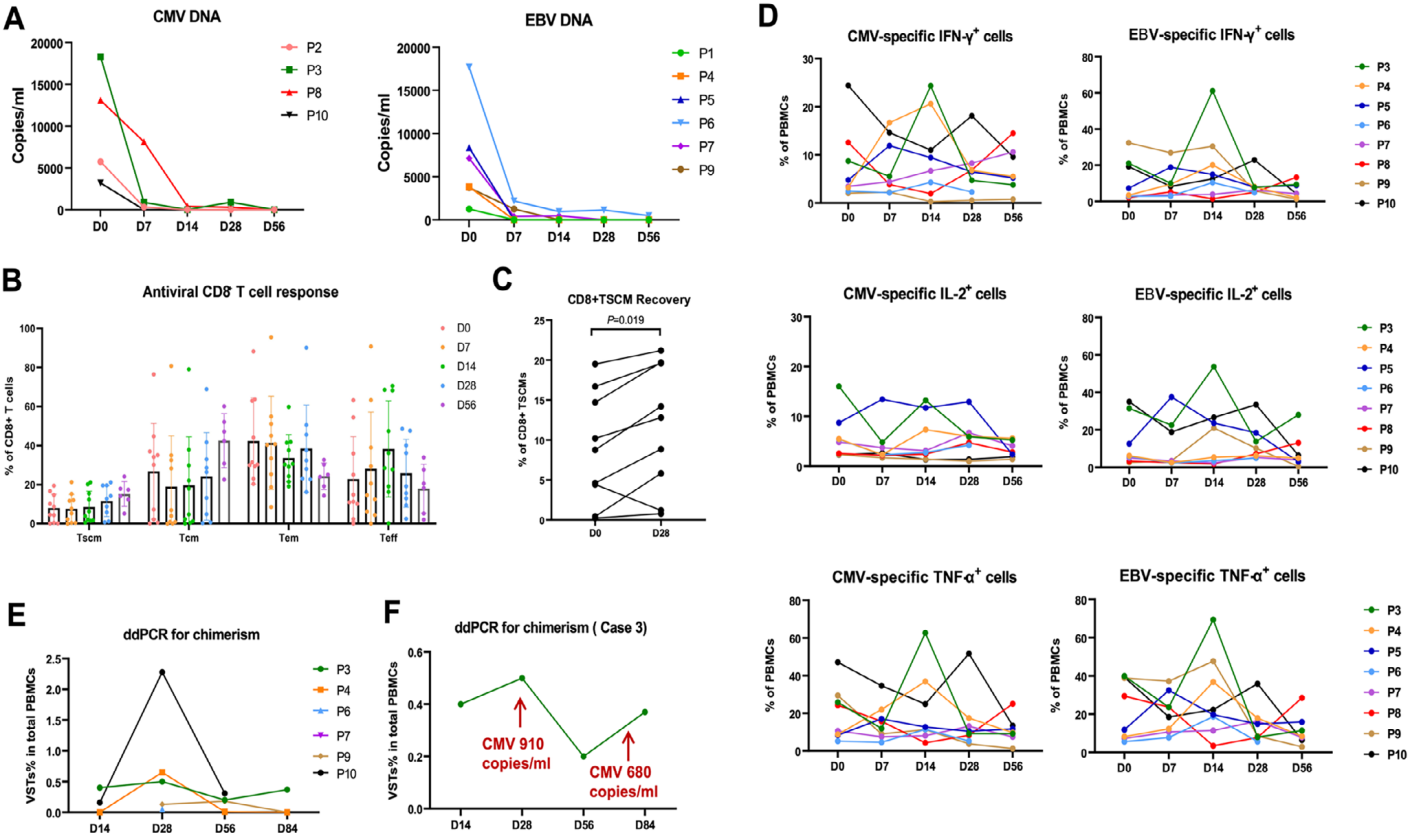
Antiviral immune response after VSTs infusion in clinical patients. A) Plasma CMV and EBV DNA load after VSTs infusion. B) Antiviral CD8 T cells response after VSTs infusion. C) CD8+TSCM recovery before and 28 days after VSTs infusion. D) Antiviral immunity response to CMV and EBV peptides by FlowSpot. E) In vivo persistence of VSTs evaluated in 6 cases and F) case 3 using digital PCR (ddPCR). The definitions of T cell subsets (TSCM, TCM, TEM, TEFF) were based on surface marker expression as follows: TSCM (CD45RA⁺CD62L⁺CD95⁺), TCM (CD45RA^−^CD62L⁺), TEM (CD45RA^−^CD62L^−^), and TEFF (CD45RA⁺CD62L^−^).

By 28 days after T cell infusion, all 10 treated patients responded to VSTs therapy, with a significant decrease in viral load (**Figure** **7**A). Of these, seven patients (70.0%) achieved a complete response (CR) and three patients (30.0%) achieved a partial response (PR). The median time for viral clearance was 7 (2–21) days after the first infusion. Among the four patients with refractory CMV viremia or CMV disease, a 100% CR rate was observed, with 3 of 4 remaining free of CMV during the observation period. However, one patient, who had received HSCT from a CMV IgG‐negative donor and had a history of refractory CMV viremia and CMV pneumonia for 2 months prior to VSTs therapy experienced transient low‐level CMV viremia at 4 and 6 weeks, followed by CMV reactivation 6 months after VSTs infusion. Subsequently, the patient received a third VSTs infusion off‐study as compassionate treatment and achieved a second CR. Of the six patients with refractory EBV‐PTLD, three achieved sustained CR without evidence of reactivation, whereas the other three achieved PR. Of the three patients with PR, two cleared EBV DNA from the plasma but had partial remission of intracranial EBV‐PTLD after two cycles of VSTs infusion.

One patient died of intracranial hemorrhage with remission in EBV‐PTLD at 59 days post VSTs infusion, while another died of viral pneumonia after clear CMV DNA viremia at 99 days, and a third patient with PR eventually died of EBV‐PTLD at 124 days after VSTs infusion. The remaining seven patients were alive during the observation period of 475 (181–598) days after VSTs therapy.

### 2.8 Antiviral Immunity Response

Peripheral PBMCs were analyzed for in vivo immune recovery before and after VSTs on days 7, 14, 28, and 56. As tested for CD8+T cell subsets via flow cytometry, CD8+TSCM increased in eight of the nine evaluable patients (Figure 7B,C) after VSTs therapy by day 28. The antiviral immune response to CMV and EBV peptides by FlowSpot also showed that virological responses were achieved in most of the treated patients (Figure 7D).

To evaluate the in vivo persistence of these third‐party VSTs in the circulation after adoptive therapy, chimerism in patient PBMCs was analyzed using ddPCR to track the VST‐specific sequences before and after infusion. Owing to limited sample availability, ddPCR could not be performed at all time points for all patients. Nevertheless, third‐party‐derived VSTs were detectable in all six recipients for at least 4 weeks (Figure 7E). In one patient with refractory CMV, VSTs persisted for at least 12 weeks after therapy and showed a notable boost following CMV reactivation (Figure 7F). These findings suggest that the infused VSTs elicited an antiviral effect, contributing to viral control.

## 3 Discussion

Third‐party virus‐specific T cells represent a promising strategy for treating viral infections in immunocompromised patients, particularly in those with post‐transplant complications.^[^ ^1^, ^3^ ^]^ Although adoptive T‐cell therapies have shown promise, their long‐term efficacy and persistence remain concerns. Recent preclinical studies have suggested that different memory T‐cell subsets contribute unequally to antiviral immunity.^[^ ^15^, ^16^, ^17^ ^]^ Among these, TSCM has emerged as a particularly attractive candidate owing to its superior self‐renewal, multipotency, and long‐term persistence. However, whether virus‐specific TSCMs exert stronger antiviral effects than TCM or TEM T cells remains unclear. In this study, we developed a novel, off‐the‐shelf, TSCM‐enriched VST product targeting both CMV and EBV and systematically evaluated its functional and mechanistic advantages. Our preclinical experiments demonstrated that TSCM exhibit moderately enhanced antiviral activity, which was attributed to their unique self‐renewal and differentiation capacities. Consistent with these findings, our phase I clinical trial showed that third‐party CMV‐ and EBV‐specific TSCM‐enriched VSTs achieved a 100% cumulative response rate with no severe adverse events, confirming their safety and therapeutic potential in allo‐HSCT recipients.

Consistent with the results of Palianina et al.,^[^ ^15^ ^]^ our product was predominantly composed of TSCMs, but with notable improvements. Compared to the 3.4‐fold expansion efficiency reported by Palianina et al., our optimized cytokine‐based protocol achieved an average 13‐fold expansion in 2 weeks, starting from as little as 2 × 10⁷ PBMCs. This represents a major manufacturing advantage because it reduces the input material requirements while yielding clinically relevant doses. Moreover, our product introduced dual‐pathogen specificity (CMV and EBV) using an off‐the‐shelf platform, broadened the therapeutic spectrum, and validated the broad antiviral potential of TSCM‐enriched VSTs.

Mechanistic insights from our transcriptomic and epigenetic analyses revealed that virus‐specific CD8⁺ TSCMs were characterized by enriched MAPK signaling and activation of the Wnt pathway, consistent with previous reports on the role of Wnt/β‐catenin signaling in maintaining TSCM populations.^[^ ^18^ ^]^ In contrast to conventional VSTs dominated by TEM subsets, our cytokine combination system (IL‐2, IL‐4, IL7, IL‐12, IL15) demonstrated superior capacity for ex vivo expansion of a high proportion of TSCM phenotypes. This may be attributed to coordinated regulatory networks involving multiple signaling axes. For example, IL‐15–mediated attenuation of mTORC1 activity has been reported as a critical mechanism for preserving the TSCM phenotype;^[^ ^20^ ^]^IL‐7 provides complementary homeostatic signals that promote weak mTORC1 activation and induce memory formation through transcriptional FOXO1 and metabolic AMPKα1 pathways;^[^ ^19^, ^21^ ^]^ and IL‐12 synergizes with IL‐7 to reduces the frequency of terminally exhausted T cells and provides immune‐activating signals mediated by STAT4 activation.^[^ ^22^, ^23^ ^]^ Together, these cytokines may create a culture environment that favors the generation and maintenance of stem‐like memories. Future investigations should focus on dissecting these intricate mechanistic interactions to optimize TSCM‐enriched cellular products for enhanced therapeutic efficacy.

Despite the growing interest in TSCMs, current studies investigating virus‐specific TSCM responses remain limited and are largely confined to clinical correlations rather than mechanistic insights. For instance, functional HCV‐specific CD4+ and CD8+TSCMs can be robustly induced through adenoviral or modified vaccinia Ankara vector‐based vaccination strategies, suggesting vaccine responsiveness and potential antiviral capacity.^[^ ^24^, ^25^ ^]^ In the context of HCV or HIV/HCV co‐infection, Lu et al. reported an increased frequency of CD8⁺ TSCMs, which negatively correlated with HCV viral load, supporting their functional role in viral control.^[^ ^26^ ^]^

Our phase I trial further demonstrated the feasibility of off‐the‐shelf CMV‐ and EBV‐specific TSCM‐enriched VSTs. In a cohort of 10 patients with refractory infections, we achieved a 100% cumulative response rate, with 70% complete responses and 30% partial responses. Notably, no de novo acute GVHD occurred despite partial HLA matching (1–2 class I alleles), which is consistent with previous reports on the safety of third‐party VSTs both by us and other groups.^[^ ^10^, ^11^ ^]^ This suggests that stringent HLA compatibility may not be an absolute requirement for effective adoptive therapy, and even limited matches can elicit robust antiviral immunity without provoking alloreactivity. However, we should note that the infused VSTs doses in our study were relatively low, and the cohort size was limited (n = 10), warranting further validation in larger studies. In addition, most patients (7/10) received VSTs infusions during the early post‐HSCT period, while still receiving cyclosporine A, which likely contributed to the low incidence of GVHD. Moreover, our data indicated that a relatively small bank of VSTs lines could potentially cover most patients, providing a practical and scalable solution for viral infections in transplant recipients. Together with our in vitro and murine experiments, these results demonstrated that TSCM‐enriched VSTs exhibited enhanced and durable antiviral activity, likely contributing to robust clinical responses. Notably, our clinical observations revealed differential response patterns: VSTs demonstrated superior efficacy against CMV infections (100% CR rate) compared with EBV‐PTLD (50% CR rate). This disparity suggests that augmentation of the infusion frequency may be required to optimize the outcomes in EBV‐PTLD cases.

Immunomonitoring provided further insights into the antiviral mechanisms of the infused TSCMs. Flow cytometric analysis revealed an expansion of CD8⁺ TSCMs in most patients, reflecting both the persistence and functional recovery of antiviral immunity following therapy. Furthermore, antiviral responses to CMV‐ and EBV‐derived peptides were observed in most patients, suggesting that VSTs enriched with TSCMs effectively restored antiviral immunity. Importantly, the VSTs persisted for at least 12 weeks after post‐infusion and expanded in vivo after CMV reactivation. Although persistent VSTs were not consistently detectable in the peripheral blood in all tested cases, similar to findings from CAR‐T studies where CAR‐T cells were rarely observed in peripheral blood but were abundantly detected in post‐treatment tissue biopsies,^[^ ^27^ ^]^ we speculate that infused VSTs may preferentially migrate and home to tissue sites. These findings will pave the way for larger clinical trials to further evaluate the therapeutic potential of VSTs in a broader patient population.

Although our in vitro and animal experiments showed that TSCMs exhibited stronger antiviral effects than TEM and TCM cells, the in vivo persistence of TSCMs after infusion needs to be further enhanced. This biological phenomenon suggests that diminished peripheral TSCM counts may reflect tissue‐specific chemotaxis. Although phenotypically stem cell‐like, TSCMs rely on chemokine receptor‐mediated trafficking to exert their functions in target tissues. Our data also confirmed that adhesion molecules, such as CXCR3 and CD27, were highly expressed in TSCMs, indicating that these cells possess strong chemotactic properties. Gattinoni et al. demonstrated that murine TSCMs express a distinct pattern of chemokine receptors, including CCR7 and CXCR3, that guide lymphoid and inflamed tissue homing.^[^ ^12^ ^]^ In contrast, Cieri et al. reported that although CD8+ TSCMs possess long‐lived potential, their migratory behavior is more constrained than that of TEM cells, possibly because of differential integrin and selectin expression.^[^ ^19^ ^]^ However, in human adoptive settings, disrupted or suboptimal chemokine gradients may impair effective migration of infused TSCMs. Thus, engineering TSCMs with optimized chemokine receptor profiles or improving the local chemokine milieu may further enhance their in vivo persistence and efficacy. Additionally, reactivation observed in one patient underscores the potential need for booster infusions or adjunct therapies. Moreover, while our in vitro cytotoxicity assays employed target cells and effector T cells that were matched at HLA‐A*0201, we recognize that the full exclusion of alloreactive responses would require broader HLA compatibility analysis and functional testing using mismatched controls. In third‐party VSTs infusion, it is particularly important to ensure antigen specificity and avoid off‐target effects. Future studies incorporating comprehensive HLA typing and alloreactivity assays are essential to further validate the specificity and safety of TSCM‐enriched products.

Although our in vitro experiments demonstrated that CD8⁺ TSCMs possessed enhanced self‐renewal and differentiation capacities compared to TCM and TEM cells, the in vivo differences were less pronounced (Figure 4H). This may reflect the limitations of the xenogeneic mouse environment, which does not fully recapitulate human TSCM biology.^[^ ^12^ ^]^ Moreover, the long‐term persistence and durable multilineage potential of TSCMs are typically revealed only after extended follow‐up or repeated antigen exposure, which may not be fully captured in short‐term murine experiments. Another limitation of our study is the lack of a direct comparison between TSCM‐enriched products and conventional VST products, which would help clarify whether the enrichment strategy confers advantages over standard approaches.

In conclusion, our study provides important mechanistic and translational insights into the role of virus‐specific CD8⁺ TSCMs. By systematically comparing the antiviral functions of TSCM, TCM, and TEM subsets and by demonstrating the feasibility, safety, and efficacy of third‐party TSCM‐enriched VSTs in post‐transplant patients with CMV or EBV reactivation, we highlight the potential of early differentiated T cells as a superior platform for adoptive antiviral therapy. Future studies with extended clinical follow‐ups and head‐to‐head comparisons with conventional VST products are essential to fully define the therapeutic advantages of TSCM‐enriched strategies.

## 4 Experimental Section

### Generation of Polyclonal CMV‐ and EBV‐Specific T Cell Products

To generate polyclonal CMV‐ and EBV‐specific T cell products, a previously published rapid approach was adopted,^[^ ^28^ ^]^ with specific optimizations to enrich TSCM content. Briefly, peripheral blood mononuclear cells (PBMCs) were isolated from heparinized peripheral blood obtained from healthy volunteers by density gradient centrifugation over lymph preparation (stem cells). A total of 2 × 10^7^ PBMCs were started and stimulated overnight with 10 µL MACS GMP T cell TransActTM (Miltenyi Biotec) and a pool of peptides covering CMV‐pp65, CMV‐IE1, EBV‐LMP2A and EBV‐EBNA1(Miltenyi Biotec, 2 µg per peptide). Cells were resuspended in 40 mL of AIM‐V medium (Gibco) supplemented with 3% growth supplements (Helios), 25 U mL^−1^ IL‐2, 30 ng mL^−1^ IL‐4, 10 ng mL^−1^ IL‐7, 10 ng mL^−1^ IL‐12, and 40 ng mL^−1^ IL‐15 and cultured for 14 days in a G‐Rex10 device (Wilson Wolf) (Figure S1A, Supporting Information). The VSTs were then resuspended in a protective solution and subjected to programmed cooling procedures for long‐term storage, which were referred to as VST products.

### Cell Lines and Virus

Raji, Raji‐pp65, and Raji‐HLA‐A*0201‐pp65‐luciferase‐GFP cells were kindly provided by Professor Lin Xin, Tsinghua University, and pp65 (complete antigen) and HLA*A0201 were lentivirus‐ transduced for stable expression.^[^ ^29^ ^]^ B95‐8 cells were purchased from ProCell Life Science and Technology (Wuhan, China). MRC‐5 human fetal lung fibroblasts were obtained from the National Infrastructure of Cell Line Resources cell bank. These cell lines have been authenticated within the last 3 years using STR profiling. All experiments were performed using mycoplasma‐free cells. AD169 is a laboratory‐adapted HCMV isolate, originally isolated from human adenoids and extensively passaged in fibroblasts. It is widely used in immunological and virology studies because of its stable growth and high titers.^[^ ^30^ ^]^ B95‐8 cells were used to generate EBV and immortalized B lymphocyte cell lines(B95‐8‐LCL) were established from PBMCs of separate HLA*A0201 healthy donors. All cell lines were maintained in RPMI‐1640 or DMEM supplemented with 10% fetal bovine serum (FBS, Gibco), L‐glutamine, and antibiotics under standard culture conditions (37 °C, 5% CO_2_).

### In Vitro Functional Assays

To ensure HLA compatibility and effective antigen presentation, the expanded VSTs used in these assays were derived from HLA‐A*0201 healthy donors seropositive for CMV and EBV. Purified virus‐specific CD8+T subsets were sorted based on CD45RA and CD62L from expanded VST products (% of CD3+CD8+CD95+ population) as follows: TSCM (CD45RA⁺CD62L⁺), TCM (CD45RA^−^CD62L⁺), TEM (CD45RA^−^CD62L^−^), and TEFF (CD45RA⁺CD62L^−^).

### Proliferation and Self‐Renewal Assays

Sorted CD8⁺ T cell subsets were labeled with 5 µM CFSE (Invitrogen) for 15 min at 37 °C and then stimulated with αCD3/CD2/CD28‐coated beads (Miltenyi Biotec) for 5 days in RPMI‐1640 medium supplemented with 10% FBS. The proliferation index and percentage of divided cells were analyzed using the FlowJo software (Tree Star). The stemness index was calculated as described by Gattinoni et al.^[^ ^12^ ^]^

### Anti‐CMV and EBV Functional Assays

MRC‐5 fibroblasts were seeded into 24‐well plates at a density of 1 × 10⁵ cells per well and cultured in complete medium. Upon reaching confluence, the cells were either infected with the HCMV strain AD169 at a multiplicity of infection (MOI) of 2 for 2 h (designated AD169‐MRC‐5) or left uninfected as a control. Cultures were maintained at 37 °C in a humidified incubator with 5% CO_2_ and monitored daily for the development of cytopathic effects for 24 h.

Immortalized B95‐8‐LCL were generated using EBV derived from B95‐8 cells and peripheral blood mononuclear cells (PBMCs) from allogeneic healthy HLA‐A*0201 donors, as described by Peng et al.^[^ ^31^ ^]^

For cytotoxicity and cytokine secretion assays, target cells—including Raji‐pp65‐HLA*A0201, EBV‐LCLs, and AD169‐MRC‐5 cells—were co‐cultured with purified CD8⁺ TSCM, CD8⁺ TCM, or CD8⁺ TEM cells at the indicated effector‐to‐target (E: T) ratios for 5 h. GolgiStop was added at 0.7 µL mL^−1^ (BD Biosciences) 1 h after the start of the assay. Cytotoxicity was assessed by measuring CD107a expression, perforin, and granzyme B levels, while cytokine production was determined by intracellular staining for IFN‐γ, TNF‐α, and IL‐2. All effector T cells used in the co‐culture assay were confirmed to be HLA‐A*0201 positive.

For apoptosis assays, AD169‐MRC‐5 or B95‐8‐LCL cells were labeled with CFSE (BD Horizon) and used as targets at an E:T ratio of 1:1 for either 24 h or 5 days. After 24 h of co‐culture, target cell apoptosis was evaluated using an Annexin V/7‐aminoactinomycin D apoptosis detection kit (Becton Dickinson), following the manufacturer's instructions. Effector T cells were analyzed for their differentiation status using CD45RA, CD62L, and CD95 markers.

To quantify the viral load, culture supernatants were collected on day 5, and HCMV and EBV DNA levels were determined using commercial nucleic acid detection kits (Liferiver, China), according to the manufacturer's protocols, using an ABI Prism 7300 qPCR system.

### In Vivo Models

For tumor infiltration studies, NOD‐*PrkdcscidIL2rgnull* (NPG) mice (6–8 weeks old, Beijing Vitalstar Biotechnology Co., Ltd.) were engrafted with Raji‐HLA‐A*0201‐pp65‐luciferase‐GFP cells via either the tail vein (8 × 10^4^ cells) or subcutaneous injection (1.5 × 10^5^ cells). Mice were randomly allocated to treatment groups, ensuring an equivalent mean tumor burden, prior to the administration of sorted CD8⁺ TSCM, CD8⁺ TCM, and CD8⁺ TEM cells. These T cell subsets were co‐infused via the tail vein with peripheral PBMCs depleted of CD3⁺ T cells (dT‐PBMCs,1 × 10^6^ cells) and supplemented with an equivalent number of CD4⁺ T cells isolated from the VSTs to provide the necessary cytokines and co‐stimulatory milieu in a 1:1:1 ratio. Tumor progression was monitored using an in vivo imaging system (Xenogen; Caliper Life Sciences, Hopkinton, MA, USA). Imaging was performed before T cell infusion and weekly thereafter. T‐cell frequencies in the peripheral blood and spleen were quantified using flow cytometry. Tumor volume was measured externally using calipers and was calculated using the following formula: tumor volume = ½ × (length) × (width)^2^. Each experiment was conducted in two independent batches, with three mice per group. For in vivo experiments, VSTs were similarly generated from HLA‐A*0201 healthy donors with confirmed CMV and EBV seropositivity.

### Bulk RNA Sequencing and Analysis

Purified CD8+TSCM, TCM, TEM, and TEFF cells were sorted from the in vitro expanded HLA‐A*0201 VST products. Then total RNA containing poly(A) tails was extracted using a RNeasy Micro Kit (QIAGEN). The RNA was then reverse transcribed into complementary DNA (cDNA) for subsequent sequencing. All sequencing was performed on a NovaSeq 6000 platform (Illumina) using paired‐end 150 base‐pair (bp) reads (2 × 150 bp cycles).

Paired‐end RNA‐seq reads were aligned to the human genome assembly (hg19) using HISAT2.^[^ ^32^ ^]^ The gene expression levels were quantified using FeatureCounts.^[^ ^33^ ^]^ Differential gene expression analysis was performed using DESeq2. Genes with an Benjamini‐Hochberg false discovery rate (FDR)‐adjusted p‐value 0.05&|log2Fold2change|>1.

### ATAC Sequencing and Data Analysis

ATAC sequencing was performed as previously described.^[^ ^34^, ^35^ ^]^ Paired‐end sequencing was performed using a NovaSeq6000 (Illumina). Raw ATAC‐seq FASTQ files from paired‐end sequencing were processed as described.^[^ ^36^ ^]^ Clean fastq files were aligned to the hg19 reference genome using Bowtie2. SAMtools was used to remove unmapped and unpaired mitochondrial reads. PCR duplicates were removed using Picard software. Reads were shifted +4bp and −5bp for positive and negative strand, respectively. Peak calling was conducted using MACS2 with a false discovery rate (FDR) q‐value threshold of 0.01. Peaks from all samples were combined to generate a union peak set, and overlapping peaks were merged using the BEDTools merge function. Read counts per peak were quantified using the BEDTools coverage command. Differentially accessible chromatin regions were identified following DESeq2 normalization using an FDR‐adjusted q‐value cutoff < 0.05.

### Patients and Clinical Study Design

We conducted a prospective clinical study involving HSCT recipients who met the following inclusion criteria: 1) patients aged 18–70 years; 2) refractory CMV and/or EBV DNA viremia or end‐organ disease; 3) receiving steroid treatment at less than 0.5 mg kg^−1^ day^−1^ of prednisolone equivalents when enrolled; and 4) ECOG score ≤3. The exclusion criteria were: 1) within 28 days post‐HSCT; 2) grade 3–4 acute graft‐versus‐host disease (aGVHD), severe chronic graft‐versus‐host disease (cGVHD), or serious organ dysfunction within 1 week prior to T cell infusion; 3) prior administration of other adoptive cellular therapies or T cell monoclonal antibodies; and 4) participation in any other clinical research related to drugs and medical devices within 28 days before enrollment. The HSCT protocols were consistent with those used in our previous studies.^[^ ^37^ ^]^ VSTs treatment was administered once every 2 weeks, with a total of two infusions. The primary endpoints were the dose‐limiting toxicity and adverse events within 42 days. Secondary endpoints included: 1) efficacy of VSTs against CMV and EBV within 28 days post‐infusion, 2) duration and expansion of VSTs after infusion, and 3) overall survival within a 6‐month post‐infusion period. To assess the virus‐specific immune reconstitution, PBMCs were collected pre‐infusion at Day 0, and post‐infusion at Days 7, 14, 28, and 56 (NCT06075927).

### Definitions

CMV and EBV DNA‐viremia were diagnosed according to published criteria, that is, CMV DNA and/or EBV DNA ≥1 × 10^3^ copies mL^−1^ were diagnosed in plasma using real‐time qPCR assay.^[^ ^38^, ^39^ ^]^ EBV‐associated post‐transplant lymphoproliferative disorders (PTLDs) were diagnosed as proven or probable based on published definitions.^[^ ^40^ ^]^ Refractory CMV infection is defined as a persistent or increased viral load after at least 2 weeks of appropriately administered antiviral therapy.^[^ ^41^ ^]^ Refractory EBV infection and/or EBV‐PTLD were defined as rituximab.^[^ ^40^, ^41^ ^]^ Complete response (CR) was defined as a decrease in CMV and EBV viral loads below the limits of assay detection in two consecutive tests, with the disappearance of all clinical signs confirmed by physical examination or imaging studies. Partial response (PR) was defined as a decrease in viral load by quantitative PCR of at least 50% from baseline or a 50% improvement in clinical signs and symptoms. For EBV‐PTLD, treatment response was defined according to the International Workshop Criteria for assessing the response to treatment in non‐Hodgkin lymphoma.^[^ ^42^ ^]^

### Statistical Analysis

Statistical analyses were performed using GraphPad Prism 8.0(GraphPad Software, Boston, USA). The Mann–Whitney U test or Wilcoxon signed‐rank test was used for comparisons between two groups, depending on whether the data were paired or unpaired. For comparisons involving more than two groups, one‐way analysis of variance (ANOVA) with the Kruskal‐Wallis test was applied. Data are presented as mean ± SEM unless otherwise specified. Statistical significance was defined as *p* < 0.05, with *p* < 0.01 and *p < 0.001 indicating increasing levels of significance.

### Ethics Statement

All animal experiments were approved by the Ethics Committee of Peking University People's Hospital (Approval No. 2020PHB067‐01) and were conducted in accordance with institutional guidelines and the National Institutes of Health Guide for the Care and Use of Laboratory Animals. Human sample collection and related experiments were approved by the Ethics Committee of Peking University People's Hospital (Approval No. 2023PHD006‐008). Written informed consent was obtained from all participants prior to sample collection.

## Conflict of Interest

The authors declare no conflict of interest.

## Author Contributions

X.‐H.C. and X.‐Y.P. contributed equally to this work. X.H. and X.Z. designed the study and acquired funding. X.C. and X.P. designed and performed experiments and analyzed data. X.C., J.X., X.P., N.S., and Y.D. performed the mouse experiments. X.C, X.P., and X.Z. wrote the manuscript. X.H. and X.Z. commented on and revised the manuscript.

## Supporting information



Supporting Information

Supporting Information

## Data Availability

The data that support the findings of this study are available from [Sequence Read Archive]. Restrictions apply to the availability of these data, which were used under license for this study. Data are available from the authors with the permission of [Sequence Read Archive].
