# Correction to “Safety and Efficacy of Adoptive Transfer of Stem Cell Memory Enriched Virus Specific T Cells against CMV and EBV”

**DOI:** 10.1002/advs.74386

**Published:** 2026-02-17

Xun‐Hong Cao, Xu‐Ying Pei, Yuan‐Yuan Zhang, Juan Xie, Jing‐Wei Tu, Zhuo‐Jun Liu, Yi‐Yang Ding, Chen‐Hua Yan, Yu‐Hong Chen, Yu Wang, Lan‐Ping Xu, Xiao‐Hui Zhang, Xiao‐Jun Huang, Xiang‐Yu Zhao. “Safety and Efficacy of Adoptive Transfer of Stem Cell Memory Enriched Virus Specific T Cells against CMV and EBV.” DOI: https://doi.org/10.1002/advs.202510288. Adv Sci (Weinh) 2026 February; 13(9):e10288

In the current published version“ 2.7. Clinical Responses and Outcomes of Off‐The‐Shelf VSTs Enriched with TSCM Against CMV/EBV Infection” section, the text

“We presented clinical data from 10 patients in three dose groups (Table 1), with four patients receiving the first dose of 1 × 10^6^ m^−^^2^, three patients receiving the second dose of 2 × 10^6^ m^−^^2^, and three patients receiving the third dose of 5 × 10^6^ m^−^^2^.” is incorrect. **The correct dosing regimen should be:** “We presented clinical data from 10 patients in three dose groups (Table 1), with four patients receiving the first dose of 1 × 10^7^ m^−^^2^, three patients receiving the second dose of 2 × 10^7^ m^−^^2^, and three patients receiving the third dose of 5 × 10^7^ m^−^^2^.”

We confirm that the dosing information presented in Table 1 (“Clinical information of VSTs recipients”) **1 × 10^7^ m^−2^, 2 × 10^7^ m^−2^, and 5 × 10^7^ m^− 2^** is correct as published.

We apologize for this error.

