# Supplementary material for: Safety and Efficacy of Adoptive Transfer of Stem Cell Memory Enriched Virus Specific T Cells against CMV and EBV
Source: Adv Sci (Weinh). 2025 Dec 8;13(9):e10288. doi: 10.1002/advs.202510288 (PMC12904015; doi:10.1002/advs.202510288)
Supplement: Supplementary file 1 — Supporting Information [file ADVS-13-e10288-s002.docx]

**Supplementary materials and methods**

**Title: Safety and Efficacy of adoptive transfer of stem cell memory enriched virus specific T cells against CMV and EBV**

Authors: Xun-Hong Cao^1^#, Xu-Ying Pei^1^#, Yuan-Yuan Zhang^1^, Juan Xie^1^, Jing-Wei Tu^2,3^, Zhuo-Jun Liu^1^, Yi-Yang Ding^1^, Chen-Hua Yan^1^, Yu-Hong Chen^1^, Yu Wang^1^, Lan-Ping Xu^1^, Xiao-Hui Zhang^1^, Xiao-Jun Huang^1,4^*, Xiang-Yu Zhao^1^*

**Integrated ATAC-seq and RNA-seq analysis**

Differentially accessible chromatin regions from ATAC-seq (annotated peaks with log2Foldchange and adjusted p values) and differentially expressed genes from RNA-seq (gene symbol, log2Fold-change, adjusted p value) were used as input. Data entries with missing gene symbols or values were removed. ATAC-seq and RNA-seq results were merged at the gene level using gene symbols. To define significant changes, thresholds of |log2Fold-change| ≥ 0.5 and adjusted p < 0.05 were applied for both datasets.

**Intracellular staining**

For phenotypic analysis, expanded T cells were fixed and permeabilized using the BD Cytofix/Cytoperm kit (BD Biosciences) following the manufacturer’s protocol, then stained with fluorochrome-conjugated antibodies targeting intracellular antibody.

**Alloreactivity assay**

To evaluate potential alloreactivity of VSTs, autologous or allogeneic PBMCs were adjusted to a concentration of 1*10^6^ cells/ml (100ul per tube) and co-cultured with VSTs (100ul per tube) at four different effector-to-target (E: T) ratios (1:1,2:1,5:1, and 10:1). A spontaneous apoptosis control group (target cells cultured alone) was included in parallel. After 4h of incubation, cells were collected and stained with propidium iodide (PI). Flow cytometry was performed to assess cytotoxic activity, defined as the percentage of PI+ cells within the CFSE-negative target cell population. VSTs prepared from three independent batches were used in this assay, and no significant cytotoxicity against allogeneic targets cells indicated minimal alloreactivity of the VSTs.

**ELISpot analysis**

IFN-γ ELISpot assays were performed using the Human IFN-γ ELISpot kit (Mabtech) according to the manufacture’s protocol. Briefly, PBMCs or VSTs were stimulated in vitro with peptides mix (CMV-pp65 and CMV-IE1,EBV-LMP2A and EBV-EBNA, Miltenyi Biotec) for 4 h at 37℃ in concentrations of 5 μg/mL peptides, and thereafter IFN-γ-releasing T cells were detected using IFN-γ ELISpot assay-detection kit (Mabtech) ，IFN-γ spots were counted by the Mabtech ASTORTM. Responses to individual viral antigens were measured by adding each peptide at a concentration of 0.6nmol separately to the culture wells. To ensure that the test data is within the optimal detection range of the instrument, PBMCs were plated at 2×10^5^ cells per well and VSTs were plated at 5×10^4^ cells per well, Negative controls consisted of unstimulated cells and all experimental SFC values have been normalized by subtracting the corresponding negative control values, activating cells with anti-CD3 antibody (0.25ul per 5*10^4^ cells) served as the positive control. Mean spot counts of replicates were calculated, and the linear regression equation (R² > 0.98) was used to calculate the equivalent spot count for 2×10⁵ cells from wells plated with 5×10⁴ cells [1]. The absolute fold expansion of CMV(EBV)-specific T cells was calculated as (post-expansion ELISPOT SFCs × total cell number after expansion) / (pre-expansion ELISPOT SFCs × total cell number before expansion).

**FlowSpot analysis**

FlowSpot protocol for detecting CMV and EBV-reactive cell-mediated immunity was described according to the published studies [2]. In brief, PBMCs were collected before and at 7, 14, 28, 56 days post-infusion, and were co-cultured with 19 CMV antigen peptides (μg/mL) (JPT peptide technologies, Germany) or 14 EBV antigen peptides (μg/mL) (JPT peptide technologies, Germany) as well as fluorescence-tagged capture beads (BD technologies, USA), which fully obtained IFN-γ/IL-2/TNF-α-secreting T-cells. As a positive control, phytohemagglutinin (PHA) inserted of viral peptides were used to stimulate T-cells. PBMCs with fluorescence-tagged capture beads was tested as negative controls. After 16 hours of stimulation, T-cell-released IFN-γ /IL-2/TNF-α was captured by flow beads and measured via flow cytometry

**VSTs persistence analysis by ddPCR**

DNA was extracted from PBMCs and VSTs sample using the DNA Extraction Kit (CATB, China) and subjected to qPCR(7500, ABI, USA) to detect 14 autosomal InDel loci (N1-1, N1-2, N1-3, N2-1, N5-4, N9-1, N11-1, N13-1, N14-1, N16-2, N21-1, N7-2, N8-1, N22-1) and screen for differences between the recipient and VSTs sample. Selected InDel loci were detected by droplet digital PCR (SG-2000, Rainsure Scientific, China). Reactions contained PCR MIX, DNA, InDel loci primers/probes. After PCR amplification, droplets were quantified (SG-2000 Reader), and VSTs specific InDel loci were used to calculate chimerism rates.


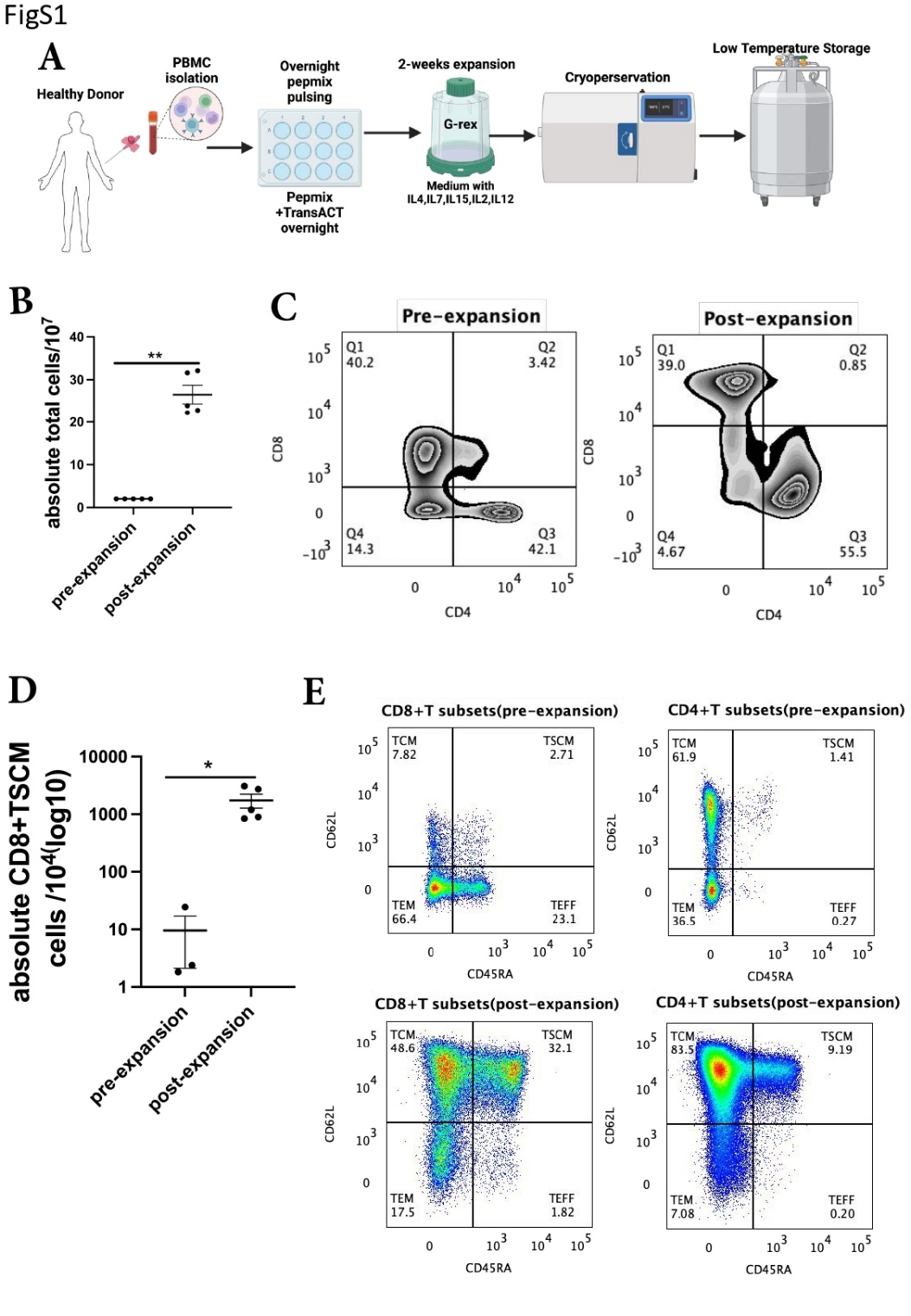


**FigureS1:** The expansion system and characteristics of VST cells. (A)The diagram of expansion system in vitro. (B) The absolute total number of cells before and post expansion after the 2-week culture period. (C)Typical flow cytometry diagram of CD4/CD8 cell distribution (of %CD3+CD95+ cells) before and after amplification. (D) Absolute counts of CD8+TSCM cells before expansion (PBMCs) and after expansion (VST products). (E)The representative flow cytometry plot of memory T cells among CD8+T cells before and after expansion.


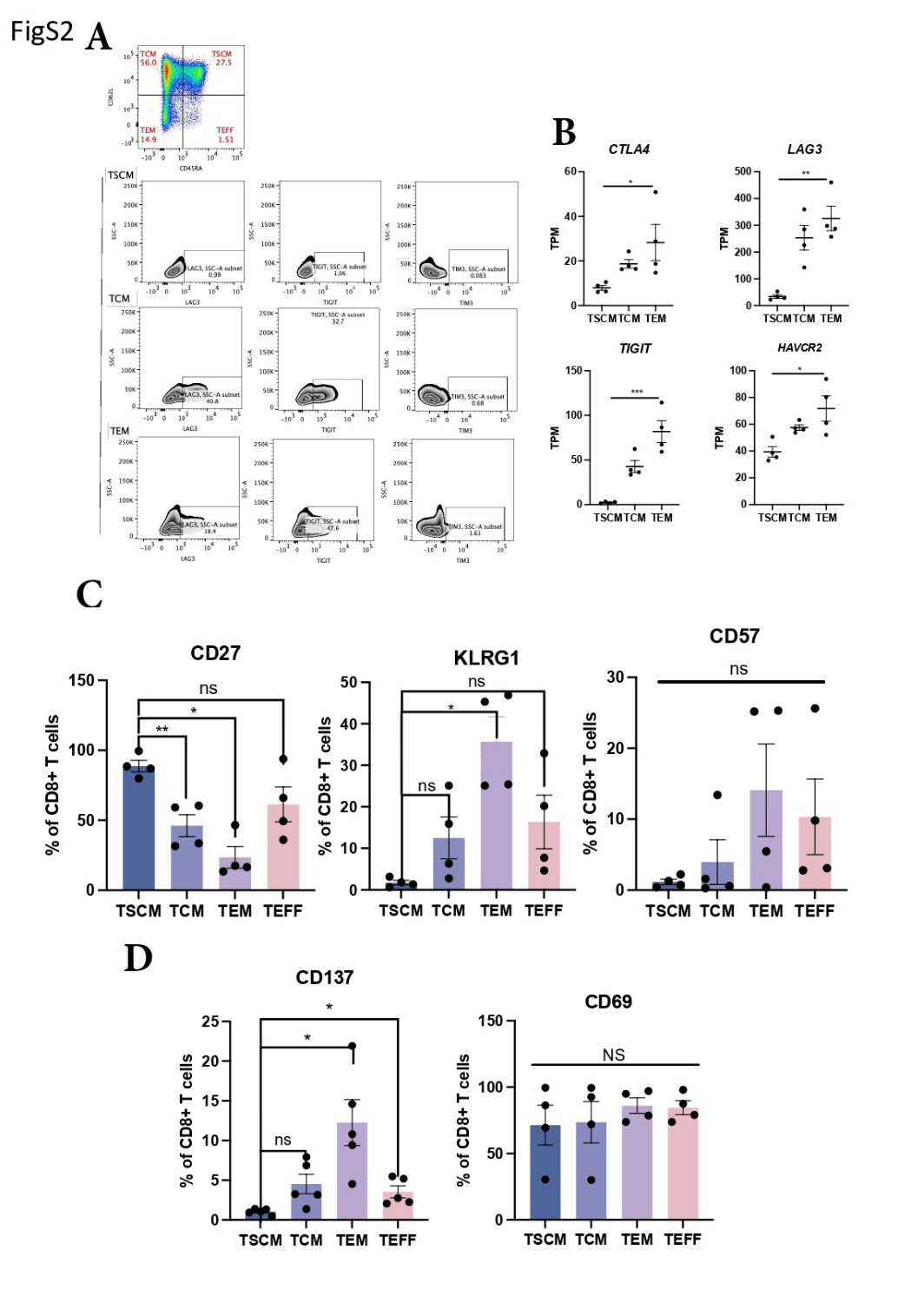


**FigureS2: The phenotypical differences among the T subgroups.** (A)Typical flow cytometry diagram of the expression of exhaustion molecules in CD8+T cell subsets; (B)The expression of CTLA4, LAG3,TIGIT and HAVCR2 transcripts in CD8+T subsets(TPM: Transcripts Per Million).(C)The expression of CD27, KLRG1 and CD57 among CD8+T cell subsets(n=4-5/group); (D) The percentages of CD137 and CD69 expression among CD8+T cell subsets(n=4-5/group, Kruskal-Wallis test).


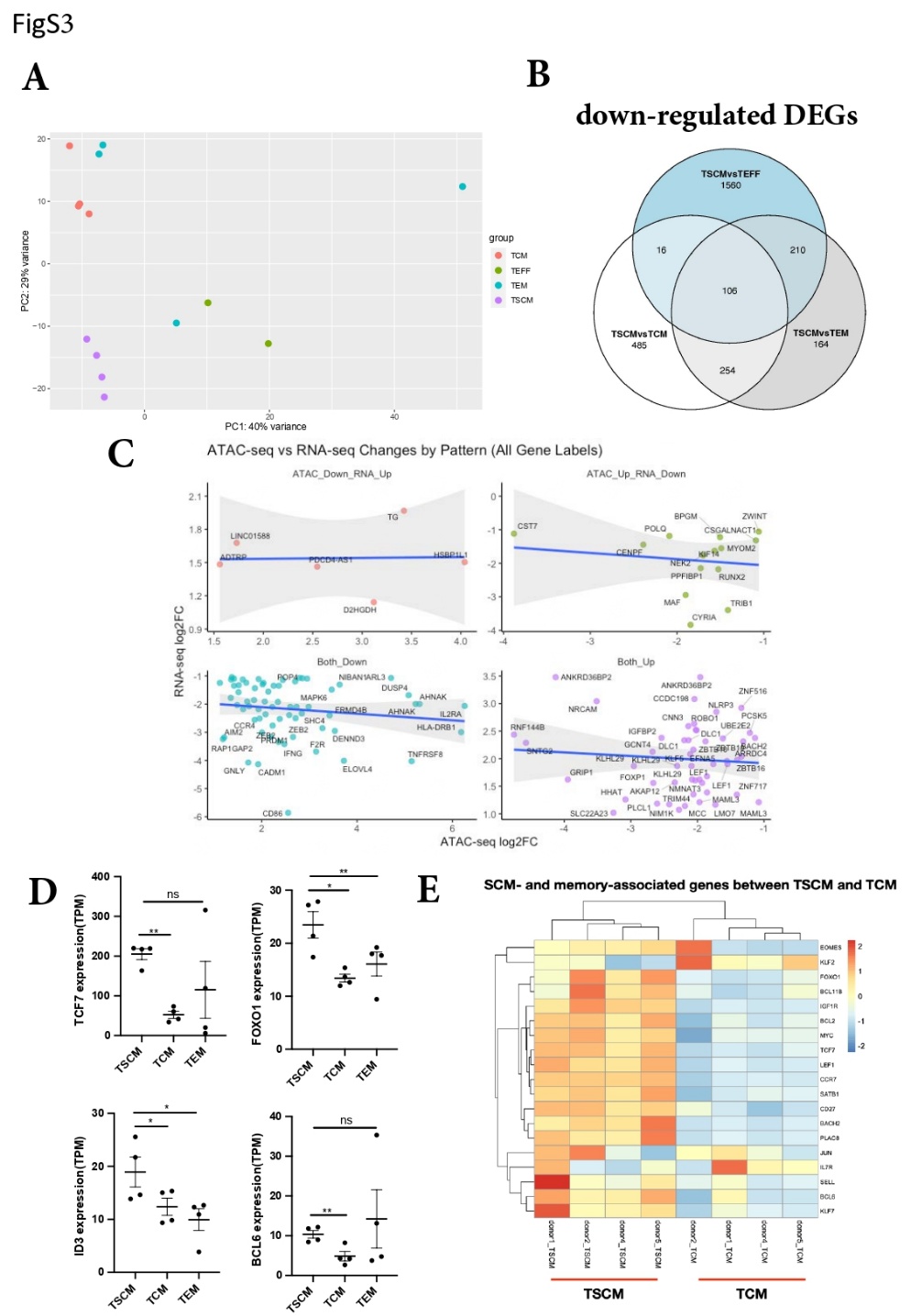


**FigureS3: The functional and molecular differences among the virus-specific T subsets.** (A)Principal component analysis shows intra- and inter-group differences among CD8+T cell subsets from RNA-seq data. (B)After comparing the two groups of TEM,TCM and TEFF with CD8+TSCM cells, the down-regulated differential genes were intersected via Venn diagram. (C) Integrated ATAC-seq and RNA-seq analysis. (D)The expression of TCF7, FOXO1, ID3 and BCL6 transcripts in CD8+T subsets. (E)Heatmap display of SCM- and memory-associated genes between CD8+TSCM and CD8+TCM cells.


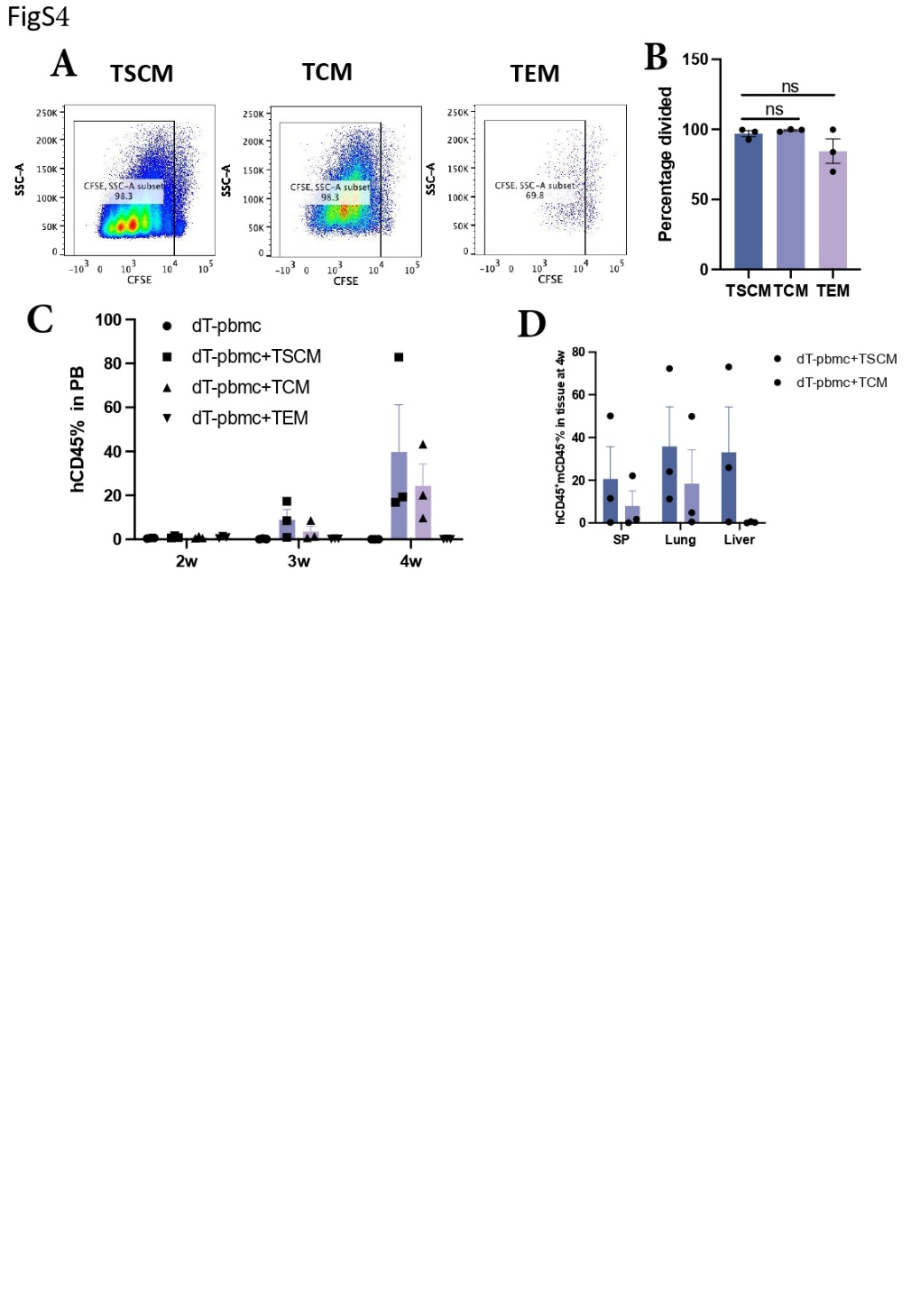


**FigureS4: The functional and distribution differences among the virus-specific T subsets.** (A-B) Diagram and ratio differences of proliferation ability among CD8+T cell subsets under the stimulation of CD2/CD3/CD28 for 5 days in vitro(n=3/group,Kruskal-Wallis test).(C)The expression of hCD45 in PB at different time points after adoptive infusion of CD8+T subsets in irradiated NPG mice(n=3/group).(D)The percentages of hCD45 expression in mouse spleen, lung and liver 4-weeks’s post T cell reinfusion;


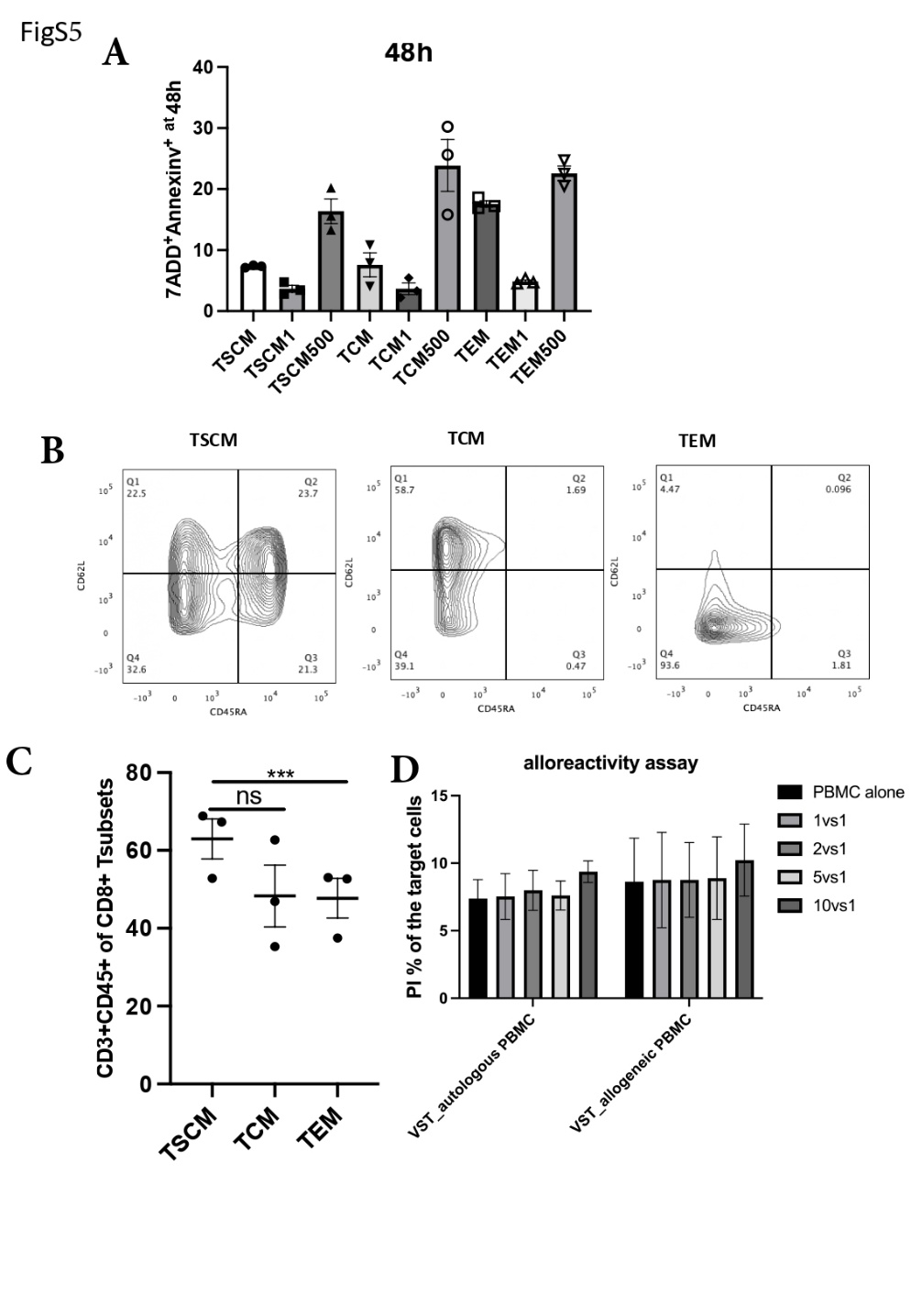


**FigureS5: The functional comparison among the CD8+T subsets.** (A) Apoptosis rate of T cells in each group after incubation of T cell subsets with AD169 virus strain for 48 h.(B)Flow cytometry diagram of T cell differentiation status after co-incubation of different CD8+T cell subsets with AD169-MRC-5 cells for 5-days.(C)The proportion of CD3+T cells after 5-day coculture with AD169-MRC-5 cells(n=3,Kruskal-Wallis test).(D) The percentage of the PI in targets cells when VSTs cocultured with autologous PBMC or allogeneic PBMC at multiple E:T ratios (1:1 to 10:1).


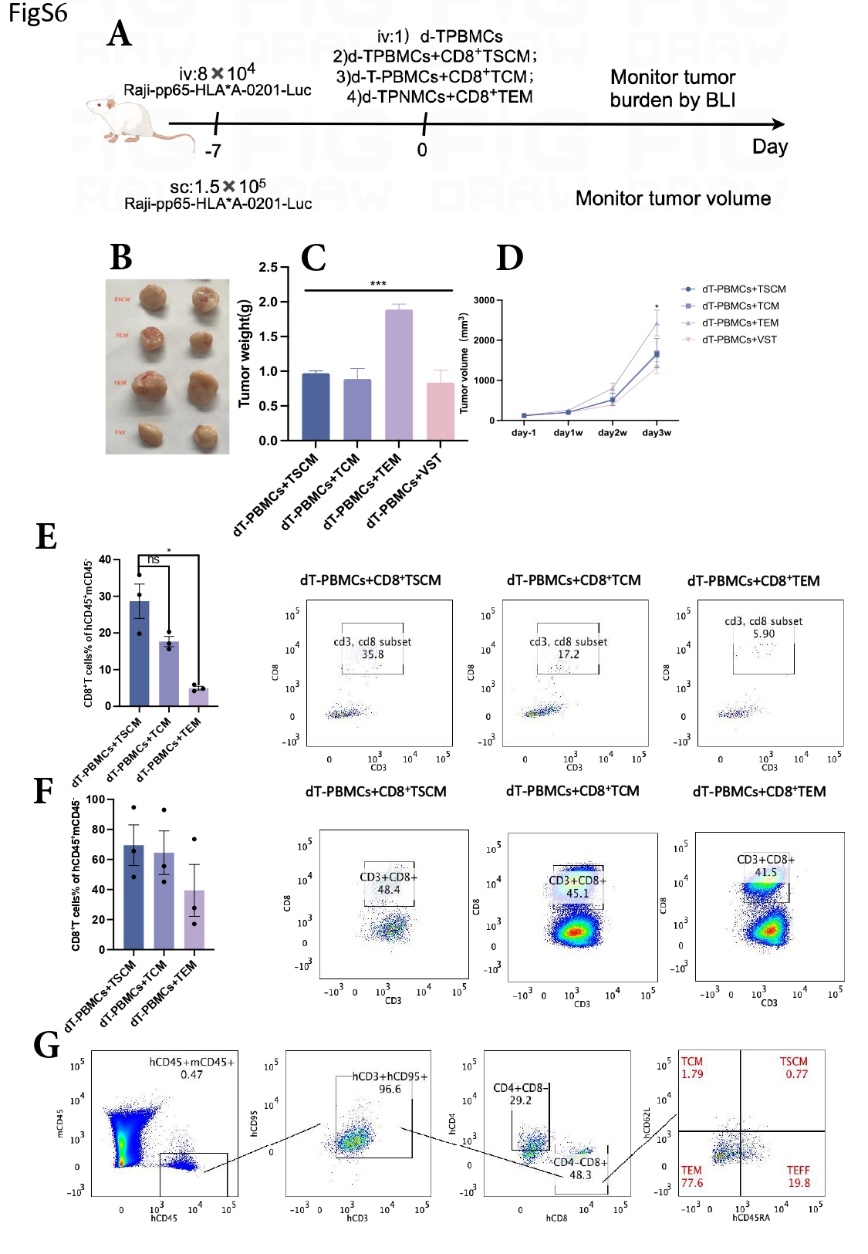


**FigureS6:** **Therapeutic effect and in vivo kinetic analysis of adoptive infusion of virus-specific CD8+T subsets on Raji-pp65-HLA*0201 tumors.** (A)Tumor-bearing pathway and cell infusion flow chart(iv: intravenous injection; sc: subcutaneous injection). (B-D) Differences in tumor size and tumor weight after infusion of different virus-specific CD8+T cell subsets in subcutaneous tumor-bearing models (n=6/group, two independent experiments). (E) Differences in the proportion of CD8+T cells in peripheral blood three-weeks after infusion of CD8+T cell subsets from intravenous-bearing model. (F) Differences in the proportion of CD8+T cells in peripheral blood three-weeks after infusion of CD8+T cell subsets from subcutaneous tumor-bearing model. (G)Flow cytometry gating strategy and representative plots of T cell subset differentiation in peripheral blood at week 3 after T cell infusion.

**Supplementary Tables**

**Table S1. Characteristics of the 10 VST products.**

| Cell line | Cryopreservation specification | Number of cryopreservation bags | HLA-A | | HLA-B | | HLA-C | | HLA-DRB1 | | HLA-DQB1 | |
| --- | --- | --- | --- | --- | --- | --- | --- | --- | --- | --- | --- | --- |
| VST001 | 2E7/bag，10 mL | 54 | 03:01 | 11:01 | 07:02 | 40:01 | 04:01 | 07:02 | 13:01 | 14:05 | 05:03 | 06:03 |
| VST002 | 2E7/bag，10 mL | 53 | 02:06 | 11:01 | 15:01 | 51:01 | 03:03 | 14:02 | 12:01 | 15:01 | 06:02 | 03:01 |
| VST003 | 2E7/bag，10 mL | 60 | 02:01 | 24:02 | 40:02 | 44:03 | 03:04 | 14:03 | 13:02 | 14:03 | 03:01 | 06:04 |
|  | 4E7/bag，10 mL | 39 |  |  |  |  |  |  |  |  |  |  |
| VST004 | 2E7/bag，10 mL | 9 | 02:06 | 11:01 | 13:01 | 40:06 | 03:04 | 08:22 | 09:01 | 15:01 | 03:03 | 06:01 |
|  | 4E7/bag，10 mL | 39 |  |  |  |  |  |  |  |  |  |  |
| VST005 | 4E7/bag，10 mL | 85 | 02:01 | 26:01 | 15:11 | 46:01 | 01:02 | 03:03 | 08:03 | 09:01 | 06:01 | 03:03 |
| VST006 | 2E7/bag，10 mL | 47 | 02:01 | 33:03 | 48:01 | 58:01 | 08:03 | 03:02 | 13:02 | 14:05 | 05:03 | 06:09 |
| VST007 | 4E7/bag，10 mL | 58 | 11:01 | 33:03 | 18:01 | 39:01 | 07:02 | 12:03 | 11:04 | 13:12 | 03:01 | 03:01 |
| VST008 | 4E7/bag，10 mL | 72 | 11:01 | 26:01 | 13:01 | 52:01 | 03:04 | 12:02 | 12:02 | 15:02 | 06:01 | 03:01 |
| VST009 | 4E7/bag，10 mL | 26 | 11:01 | 24:02 | 40:01 | 40:02 | 03:04 | 15:02 | 04:10 | 15:01 | 06:02 | 04:02 |
| VST010 | 2E7/bag，10 mL | 11 | 02:01 | 02:06 | 15:18 | 40:06 | 07:04 | 08:22 | 04:01 | 04:05 | 03:01 | 04:01 |

**Table S2. HLA typing of VST products, patients and stem cell donor.**

| **Patient No** |  | **HLA-A** | | **HLA-B** | | **HLA-C** | | **HLA-DRB1** | | **HLA-DQB1** | |
| --- | --- | --- | --- | --- | --- | --- | --- | --- | --- | --- | --- |
| 1 | VST-1 | 03:01 | 11:01 | 07:02 | 40:01 | 04:01 | 07:02 | 13:01 | 14:05 | 05:03 | 06:03 |
|  | Patient | 03:01 | 02:06 | 46:01 | 48:01 | 07:02 | 08:01 | 09:01 | 11:01 | 03:01 | 03:03 |
|  | Stem cell donor | 02:06 | 11:01 | 40:01 | 48:01 | 07:02 | 08:01 | 11:01 | 12:02 | 03:01 | 03:01 |
| 2 | VST-1 | 03:01 | 11:01 | 07:02 | 40:01 | 04:01 | 07:02 | 13:01 | 14:05 | 05:03 | 06:03 |
|  | Patient | 02:01 | 11:01 | 40:01 | 40:01 | 07:02 | 07:02 | 08:03 | 09:01 | 03:03 | 06:01 |
|  | Stem cell donor | 11:01 | 11:01 | 40:01 | 40:01 | 03:04 | 07:02 | 09:01 | 09:01 | 03:03 | 03:03 |
| 3 | VST-3 | 02:01 | 24:02 | 40:02 | 44:03 | 03:04 | 14:03 | 13:02 | 14:03 | 03:01 | 06:04 |
|  | Patient | 02:01 | 24:02 | 52:01 | 54:01 | / | / | 04:05 | 15:02 | / | / |
|  | Stem cell donor | 02:01 | 24:02 | 40:229 | 54:01 | 01:02 | 03:04 | 04:05 | 12:01G | 03:01 | 04:01 |
| 4 | VST-1 | 03:01 | 11:01 | 07:02 | 40:01 | 04:01 | 07:02 | 13:01 | 14:05 | 05:03 | 06:03 |
|  | Patient | 03:01 | 11:02 | 07:02 | 27:04 | 07:07 | 12:02 | 01:01 | 08:09 | 04:02 | 05:01 |
|  | Stem cell donor | 03:01 | 30:01 | 07:02 | 13:02 | 06:02 | 07:67 | 01:01 | 07:01 | 02:02 | 05:01 |
| 5 | VST-2 | 02:06 | 11:01 | 15:01 | 51:01 | 03:03 | 14:02 | 12:01 | 15:01 | 06:02 | 03:01 |
|  | Patient | 02:06 | 33:03 | 40:01 | 46:01 | 01:02 | 15:02 | 14:54 | 15:01 | 05:03 | 06:02 |
|  | Stem cell donor | 02:06 | 33:03 | 37:01 | 46:01 | 01:02 | 06:02 | 10:01 | 14:54 | 05:01 | 05:03 |
| 6 | VST-2 | 02:06 | 11:01 | 15:01 | 51:01 | 03:03 | 14:02 | 12:01 | 15:01 | 06:02 | 03:01 |
|  | Patient | 24:02 | 11:01 | 13:01 | 51:01 | 14:07 | 15:01 | 03:04 | 01:02 | 05:03 | 06:02 |
|  | Stem cell donor | 24:02 | 11:01 | 13:01 | 35:01 | 14:07 | 08:03 | 03:04 | 03:03 | 05:03 | 06:01 |
| 7 | VST-3 | 02:01 | 24:02 | 40:02 | 44:03 | 03:04 | 14:03 | 13:02 | 14:03 | 03:01 | 06:04 |
|  | Patient | 02:01 | 33:03 | 44:03 | 27:05 | 14:03 | 02:02 | 13:02 | 04:04 | 06:04 | 03:02 |
|  | Stem cell donor | 02:01 | 33:03 | 44:03 | 15:11 | 14:03 | 03:03 | 13:02 | 09:01 | 06:04 | 03:03 |
| 8 | VST-1 | 03:01 | 11:01 | 07:02 | 40:01 | 04:01 | 07:02 | 13:01 | 14:05 | 05:03 | 06:03 |
|  | Patient | 11:01 | 33:03 | 46:01 | 58:01 | 01:02 | 03:02 | 03:01 | 15:01 | 02:01 | 06:01 |
|  | Stem cell donor | 11:01 | 11:01 | 35:01 | 46:01 | 01:02 | 03:03 | 11:01 | 15:01 | 03:01 | 06:01 |
| 9 | VST-3 | 02:01 | 24:02 | 40:02 | 44:03 | 03:04 | 14:03 | 13:02 | 14:03 | 03:01 | 06:04 |
|  | Patient | 02:01 | 02:07 | 15:11 | 46:01 | 01:02 | 03:03 | 09:01 | 09:01 | 03:03 | 03:03 |
|  | Stem cell donor | 02:01 | 02:07 | 46:01 | 46:01 | 01:02 | 01:02 | 09:01 | 09:01 | 03:03 | 03:03 |
| 10 | VST-2 | 02:06 | 11:01 | 15:01 | 51:01 | 03:03 | 14:02 | 12:01 | 15:01 | 06:02 | 03:01 |
|  | Patient | 11:01 | 02:06 | 15:01 | 57:01 | 03:03 | 06:02 | 04:06 | 07:01 | 03:03 | 03:02 |
|  | Stem cell donor | 26:01 | 02:06 | 15:01 | 07:02 | 03:03 | 07:02 | 04:06 | 01:01 | 05:01 | 03:02 |

**Table S3:** **Adverse events after adoptive infusion of VSTs in clinical trial.**

| **Patient No.** | **Dose Group** | **CRS** | **ICANS** | **aGVHD** | **WBC↓** | **ANC↓** | **HGB↓** | **PLT↓** | **Treatment-related hematologic toxicity** | **Organ dysfunction** |
| --- | --- | --- | --- | --- | --- | --- | --- | --- | --- | --- |
| **1** | Dose1 | 0 | 0 | 0 | 2 | 2 | 2 | 2 | 0 | 0 |
| **2** | Dose1 | 0 | 0 | Prior grade 1 aGVHD and keep stable after VST therapy | 3 | 3 | 2 | 3 | 0 | 0 |
| **3** | Dose1 | 0 | 0 | Prior grade 2 aGVHD and keep stable after VST therapy | 0 | 0 | 2 | 1 | 0 | 0 |
| **4** | Dose1 | 0 | 0 | 0 | 3 | 3 | 1 | 2 | 0 | 0 |
| **5** | Dose2 | 0 | 0 | 0 | 0 | 0 | 2 | 4 | 0 | 0 |
| **6** | Dose2 | 0 | 0 | 0 | 1 | 0 | 3 | 3 | 0 | 0 |
| **7** | Dose2 | 0 | 0 | 0 | 0 | 0 | 3 | 2 | 0 | 0 |
| **8** | Dose3 | 0 | 0 | 0 | 0 | 1 | 3 | 4 | 0 | 0 |
| **9** | Dose3 | 0 | 0 | 0 | 2 | 2 | 2 | 4 | 0 | 0 |
| **10** | Dose3 | 0 | 0 | 0 | 1 | 1 | 3 | 2 | 0 | 0 |

**Abbreviations:** CRS:Cytokine release syndrome;ICANS:Immune effector cell-associated neurotoxicity syndrome; aGVHD: Acute graft versus host disease; WBC: White blood cell; ANC: Absolute neutrophil count; HGB:Hemoglobin;PLT:Platelet.

**Table S4. Antibody information.**

| **Specificity** | **Flurochrome** | **Clone** | **Vendor** |
| --- | --- | --- | --- |
| CCR7 | PE | 150503 | BD |
| CXCR3 | Percp-Cy5.5 | [G025H7](https://www.biolegend.com/en-gb/search-results?Clone=G025H7) | Biolegend |
| CD62L | PE-Cy7 | [MEL-14](https://www.biolegend.com/en-gb/search-results?Clone=MEL-14) | Biolegend |
| CD95 | APC | DX2 | Biolegend |
| CD27 | BV421 | M-T271 | Biolegend |
| CD45RA | FITC | HI100 | Biolegend |
| CTLA4 | PE | [BNI3](https://www.biolegend.com/nl-be/search-results?Clone=BNI3) | Biolegend |
| CD95 | Percp-Cy5.5 | [DX2](https://www.biolegend.com/en-us/search-results?Clone=DX2) | Biolegend |
| TIM3 | APC | [B8.2C12](https://www.biolegend.com/en-ie/search-results?Clone=B8.2C12) | Biolegend |
| CD8 | APC-R700 | SK1 | BD |
| CD4 | APC | RPA-T4 | BD |
| LAG3 | BV421 | 11C3C65 | Biolegend |
| TIGIT | BV605 | A15153G | Biolegend |
| CD62L | PE | DREG-5 | BD |
| CD8 | PE-Cy7 | SK1 | Biolegend |
| CD4 | APC-R700 | RPA-T4 | BD |
| CD69 | APC-Cy7 | FN50 | Biolegend |
| KLRG1 | BV421 | 2F1/KLRG1 | Biolegend |
| CD3 | BV510 | [OKT3](https://www.biolegend.com/nl-be/search-results?Clone=OKT3) | Biolegend |
| CD57 | BV605 | NK-1 | Biolegend |
| CD137 | PE | 4B4-1 | Biolegend |
| CD3 | APC-R700 | OKT3 | Biolegend |
| CD4 | APC-Cy7 | SK3 | BD |
| CD8 | BV421 | RPA-T8 | BD |
| CD127 | BV510 | A019D5 | Biolegend |
| CD45RA | BV605 | HI100 | Biolegend |
| CD107a | PE | H4A3 | BD |
| Perforin | APC-Cy7 | dG9 | Biolegend |
| GranzymeB | BV421 | QA18A28 | Biolegend |
| CD8 | BV510 | SK1 | Biolegend |
| mouse CD45 | Percp | 30-F11 | Biolegend |
| human CD45 | APC-Cy7 | 2D1 | Biolegend |
| IL2 | BV421 | MQ1-17H12 | Biolegend |
| IFNγ | BV510 | 4S.B3 | Biolegend |
| TNF-α | BV605 | MAb11 | Biolegend |

**Table S5. Materials used in the VSTs expansion.**

| **Materials** | **Source** | **Product Number** |
| --- | --- | --- |
| ELISpot Pro: Human IFN‑γ (ALP) | Mabtech | 3420-2APW-10 |
| PepTivator® CMV pp65 | Miltenyi Biotec | 130-097-727 |
| PepTivator® CMV IE-1 | Miltenyi Biotec | 130-093-493 |
| PepTivator® EBV LMP2A | Miltenyi Biotec | 130-093-615 |
| PepTivator® EBV EBNA | Miltenyi Biotec | 130-093-614 |
| IL2 | SL PHARM | S19991010 |
| IL4 | T&L Biotec | GMP-TL509 |
| IL7 | PeproTech | GMP200-07 |
| IL12 | T&L Biotec | GMP-TL508 |
| IL15 | T&L Biotec | GMP-TL509 |
| CTS AIM V Medium | Thermo | 0870112DK |
| UltraGRO™-Advanced | Helios | HPCFDCRL50 |
|  |  |  |

**Reference**

[1] Dulovic, A., Rabsteyn, A., Remppis, J., Gentzcke, I. K., Mueller, J., Tuecks, N., ... & Renk, H. (2025). Longitudinal analysis of humoral and cellular immunity in SARS-CoV-2 exposed families. Scientific reports, 15(1), 26041.

[2] Ye, Q., Wang, J., Chen, M., et al.2023.Interferon-gamma FlowSpot assay for the measurement of the T-cell response to cytomegalovirus. Heliyon, 9(6).
